# Engagement Methods in Brain Tumor Genomic Research: Multimethod Comparative Study

**DOI:** 10.2196/68852

**Published:** 2025-08-21

**Authors:** Matthew DeCamp, Juliana G Barnard, Carly Ritger, Laura J Helmkamp, Anowara Begum, Sandra Garcia-Hernandez, Rudy Fischmann, Nestelynn Gay, Ricardo Gonzalez-Fisher, Kevin C Johnson, Lindsay A Lennox, Guy R Lipof, Jasmyn Ostmeyer, Ifeoma Perkins, Laura Pyle, Liz Salmi, Talia Thompson, Elizabeth B Claus, Roel Verhaak, Bethany M Kwan

**Affiliations:** 1 Center for Bioethics & Humanities School of Medicine University of Colorado Anschutz Medical Campus Aurora, CO United States; 2 Division of General Internal Medicine School of Medicine University of Colorado Anschutz Medical Campus Aurora, CO United States; 3 Adult & Child Center for Outcomes Research & Delivery Science School of Medicine University of Colorado Anschutz Medical Campus Aurora, CO United States; 4 Department of Pediatrics School of Medicine University of Colorado Anschutz Medical Campus Aurora, CO United States; 5 Low Grade Glioma Registry Research Advisory Council New Haven, CT United States; 6 Department of Neurosurgery School of Medicine Yale University New Haven, CT United States; 7 Department of Emergency Medicine School of Medicine University of Colorado Anschutz Medical Campus Aurora, CO United States; 8 Department of Pathology School of Medicine University of Colorado Anschutz Medical Campus Aurora, CO United States; 9 Department of Medicine Beth Israel Deaconess Medical Center Boston, MA United States; 10 School of Public Health Yale University New Haven, CT United States; 11 Department of Neurosurgery Brigham and Women's Hospital Boston, MA United States; 12 Department of Neurosurgery Amsterdam University Medical Center Amsterdam The Netherlands

**Keywords:** engagement, brain cancer, brain tumor, low-grade glioma, research advisory council, social media

## Abstract

**Background:**

Engaging patients, care partners, and others in research planning and conduct is increasingly valued. However, identifying the most effective ways to do so remains a challenge.

**Objective:**

This study aimed to evaluate participation and participant experience using 3 engagement methods with the Low-Grade Glioma (LGG) Registry’s Optimizing Engagement in Discovery of Molecular Evolution of Low-Grade Glioma (OPTIMUM) project, part of the National Cancer Institute’s Participant Engagement and Cancer Genome Sequencing Network.

**Methods:**

We evaluated LGG Registry research advisory council (RAC) meetings, Twitter (now known as X), and Facebook discussions across 4 engagement activities with each group. Researchers recorded discussions and performed qualitative content analysis to evaluate differences in the nature of interactions and recommendations for promoting trust and participation in LGG Registry research. Participants completed experience surveys after engagements 1 and 4 (Public and Patient Engagement Evaluation Tool, Research Engagement Survey Tool, Trust in Medical Researchers Scale, and Patient Engagement in Research Scale).

**Results:**

RAC engagements involved 25 unique participants representing diverse backgrounds; tweet chats and Facebook discussions had 197 and 133 participants, respectively. Qualitative findings highlighted differences in the nature of interactions (eg, communication styles and types of information shared) across groups, but there was general agreement around recommendations for promoting participation in genomic research. Postengagement surveys (n=52 in ipostengagement activity 1; n=40 in postengagement activity 4) showed patterns suggesting a more positive experience overall for the RAC.

**Conclusions:**

Advisory councils and social media engagement methods have advantages and disadvantages. Advisory councils provide consistent interactions with the same individuals and clear procedures. Despite theoretically broader reach, social media engagement may yield less diverse perspectives. The LGG Registry aims to use RAC and social media engagement methods to promote diverse perspectives and maintain consistent interactions.

## Introduction

### Background

Engagement of patients, families, and others has become essential to research, from study conception to dissemination [1-6]. An increasing body of evidence shows that engagement can positively influence the research process—shaping the research questions asked, improving research conduct (eg, recruitment, retention, and data collection), guiding the return of results, and more—all in ways that center patient and community perspectives [7-12]. Cancer and cancer genomics research are no exception [13]. Patients have a long history of advocacy and engagement in cancer research [14,15]. Engagement is thought to be critical to promoting representativeness, recruitment, retention, and trust (understood broadly to mean participants can rely on the research enterprise to protect their interests) in genomics research [16]. For instance, engagement has the potential to improve research relevance, promote recruitment and retention, enhance dissemination, and increase diversity among research participants [17].

A recent review involving major cancer research and cancer genomics programs (such as the National Cancer Institute [NCI] Cancer Moonshot Initiative’s Participant Engagement and Cancer Genome Sequencing [PE-CGS] Network) called for measuring effectiveness and comparing methods of engagement in cancer genomics research [18]. Despite progress in the measurement of engagement effectiveness [9,19-25], comparative effectiveness research on discrete engagement methods (eg, advisory panel methods, social media–based engagement discussions, and community engagement studios) is sparse and tends to focus on a single aspect of a method (eg, group composition or online vs in-person modalities) [26,27].

Understanding the most effective, efficient, and patient-centered methods of engagement may be particularly important for rare cancers, including adult lower-grade gliomas [28]. Although rare (affecting <1 per 100,000 people in the United States), they represent up to 20% of malignant brain tumors, affect relatively young adults, and are associated with high morbidity and mortality. Genomics research promises to provide new insights into low-grade glioma (LGG) diagnosis and treatment, including understanding the many potential genomic variations in tumor types [29-31]. Because lower-grade gliomas are rare, research must often recruit from multiple geographic locations, with variability in social, economic, technological, and clinical contexts [32]. This geographically dispersed community creates challenges for research—yet presents an opportunity for studying engagement methods.

The International Low-Grade Glioma Registry (LGG Registry) was established in 2016 at Yale University to conduct genomic and epidemiological research into risk factors and outcomes for LGG. Recently, a study of the molecular evolution of LGG, termed Optimizing Engagement in Discovery of Molecular Evolution of Low-Grade Glioma (OPTIMUM) and focused on persons with recurrent LGG in the LGG Registry, was funded by the NCI as part of the Cancer Moonshot Effort and PE-CGS network [33].

To date, the LGG Registry includes more than 700 registrants from around the world. Most participants define themselves as White, of non-Hispanic ancestry, and of relatively high educational and economic status. To reduce disparities in access or willingness to engage in LGG research, the LGG Registry has developed partnerships with people living with LGG, care partners, and experts [34,35]. As it remains unclear which engagement methods are the most effective, the LGG Registry explored 3 methods for engagement of the LGG community in genomic research: a research advisory council (RAC) and online discussions via Facebook (Meta Platforms, Inc) and Twitter (X Corp) social media.

### Objectives

The purpose of this study was to compare engagement processes and outcomes among 3 methods of engaging people living with LGG, their care partners, clinicians, researchers, and others in the planning, conduct, and dissemination of genomic research.

## Methods

### Study Design and Context

Funded as part of the NCI’s PE-CGS Network, OPTIMUM aims to enroll into the LGG Registry people diagnosed with LGG who have had 2 or more surgeries for glioma. OPTIMUM’s Engagement Optimization Unit—a required PE-CGS center component—aims to identify effective and feasible strategies for engaging people with LGG and others in the planning, conduct, and dissemination of genomic research. The OPTIMUM engagement optimization unit’s primary goal is to identify effective strategies for engaging the LGG community in LGG genomics research to inform LGG Registry recruitment, data collection procedures, and the return of results. We conducted an exploratory quasi-experimental multimethod study to compare 3 engagement methods; participants were not randomly assigned to the engagement method (but instead had voluntarily chosen their assignment), but we did structure engagement activities to be as similar as possible. Our level of assessment was the engagement method itself. We used project tracking documents, audio recordings, data gathered from social media platforms, and surveys to evaluate reach, engagement experience, and trust in research. Permission was requested and granted for publishing direct quotes reported in this paper from identifiable individuals.

### Comparator Engagement Methods

An engagement method refers to “a set of tools, techniques, and processes that are used to enact all of the ‘high-level’ purposes of engagement: identify and convene partners, create reciprocal relationships (level the playing field), engage in bi-directional communication, elicit perspectives, and make decisions over time and in partnership” [36]. We compared 3 engagement methods for building relationships and gathering community input to inform research [37-40]. Engagement methods included facilitated discussions with the LGG Registry’s RAC; “tweet chats” in collaboration with the #BTSM (brain tumor social media) community (established in 2012; monthly tweet chats started in 2013) on Twitter; and interactive Facebook posts with the Oligodendroglioma/LGG Warriors (henceforth “Warriors”) private Facebook group (established in 2013), which includes primarily people living with LGG. Each engagement method involved 4 parallel engagement activities with each group, in the form of interactive discussions facilitated by the research team about topics relevant to the conduct of LGG genomics research.

Both social media groups existed before this project and were established by members of the brain tumor community, not the research team (Table 1). The RAC was established by the research team as part of the OPTIMUM project to inform optimization of LGG Registry recruitment, enrollment, and return of results strategies. RAC members were originally recruited in early 2022 from research team personal contacts, the LGG Registry contact list, and social media. The RAC consists of 25 people (including 19 people with LGG) purposefully selected to represent a range of community and scientific perspectives and to be demographically diverse (Multimedia Appendix 1). The RAC met once for an introductory call in February 2022 before the structured engagement activities described in the subsequent sections. Our overall goal was to minimize differences in how each parallel engagement activity was conducted across groups. All 3 methods were established at the time of our study; that is, the RAC, “tweet chats” with #BTSM, and Facebook “topic of the day” discussions were existing methods of engagement. First, for each engagement method, we held facilitated discussions on the same four topics: (1) trust and benefits of genomic research, (2) registry recruitment, (3) registry data collection, and (4) return of results. All engagement activities occurred during March through September 2022. Second, for each topic, we developed a facilitator’s agenda with near-identical prompts tailored to the engagement modality and population (Multimedia Appendix 2). There were typically 3 to 4 discussion prompts for each topic, aligned with 4 orienting research ethics concepts: autonomy, privacy, ownership, and relevance.

It is important to note the differences that may exist between these 3 methods that may create differences in the experience of participating in a given engagement activity. For instance, the Facebook Warriors group responded to prompts asynchronously, whereas the RAC and Twitter engagement activities occurred synchronously; this was done to respect the existing structure of the Warriors group, which included a “topic of the day” with most responses written within 24 to 48 hours. In addition, the free-flowing, unpredictable nature of these semistructured group discussions meant that impromptu prompts occurred in response to the discussion and were thus not the same across methods (as would be the case if we had used rigidly structured engagement activities). To facilitate the discussion, facilitators for each engagement activity prompted participants to clarify in their answers which prompt they were addressing in their answer.

RAC members consented to audio recording to allow for analysis. Tweet chats and Facebook posts included “transparency notices” indicating that the content of the discussions would be used to inform research priorities for the LGG Registry and that anyone who did not wish to be included in the analysis should not participate.

**Table 1:** Distinguishing features of comparator engagement methods.

Engagement features	Research advisory council virtual meetings	Facebook O/LGG^a^ Warriors group chats	#BTSM^b^ Twitter community tweet chats
Description of engagement activities	Synchronous 1-hour video conference meetings over Zoom (Zoom Communications, Inc). Brief presentation of the topic or issues, followed by 3-4 breakout rooms with moderated live discussion using group facilitation techniques following a structured agenda with prompts.	A series of 4 Facebook posts from the LGG team, 1 per day over the course of a week, in a private Facebook group. Posts included brief prompts, polls, links to external content, or graphics inviting commentary. Group members react asynchronously, typically in the 24-48 hours after the post is made.	Publicly available, 1-hour–long synchronous discussions on Twitter, promoted in advance and hosted by existing social media community leaders with the #BTSM community Participants introduced themselves, and every 15 minutes, a new topic was introduced by a host. Participants tweet in response during the 1-hour chat.
Participants	25 members: 19 people with LGG, 1 care partner, 1 clinician, 2 regulatory experts, 1 advocacy organization representative, and 1 genetics expert	Open invitation to members of the O/LGG Warriors group 133 unique participants (58, 17, and 88 participants in Facebook posts 1, 2, and 4c, respectively)	Open invitation to Twitter users; #BTSM, @gliomaregistry, and @NBTStweets followers 197 unique participants (72, 37, 81, and 77 participants in tweet chats 1-4, respectively)
Platform	Zoom web conferencing	Facebook social media platform	Twitter social media platform
Recruitment	Email with a link to an interest form sent to 447 people living with LGG, care partners, clinicians, researchers, and others involved in the LGG Registry	Group administrator for the O/LGG Warriors group (>3100 members) shared posts explaining the process and inviting group members to participate.	Partnership with the #BTSM community. To advertise tweet chats, the #BTSM community leaders posted from their accounts and the @BTSMchat Twitter account (with >3200 followers). These posts included details about the chat topics, dates and times, and special guests.
Leaders	Brain cancer expert patient (LS) and researchers	O/LGG Warriors group administrator (NG) and researchers	#BTSM social media organizers (LS and others) and researchers
Duration and frequency	Monthly 1-hour meetings (March to June 2022)	Semimonthly series of 4 “Topic of the Day” posts over 1 week (April, June, and September 2022)	Semimonthly 1-hour tweet chats following promotional tweets leading up to the chat (March, May, July, and September 2022)
Data source	Detailed meeting notes and recordings	PDFs of posts and comments	Symplur transcripts

^a^O/LGG: Oligodendroglioma/Low-Grade Glioma.

^b^#BTSM: brain tumor social media.

^c^“Engagement 3” on Facebook was not included in our analysis, as it was not intended to be part of the study. Engagement 3 used different procedures designed to assess whether mentioning research was decreasing willingness to participate, and participants were not alerted to the potential for their responses to be included in research analyses. Therefore, results present data from engagements 1, 2, and 4.

### Engagement Activity Participation

We manually tracked attendance in RAC meetings. Tweet chat participation data were generated using Symplur, a health care social media analytics company. Symplur provides downloadable spreadsheets with the total number of Twitter accounts that tweeted a particular hashtag in a given time frame, the number of tweets and mentions for each account, user-reported location, and Symplur-identified stakeholder category for each participating account. We manually counted the number of unique individuals participating (commenting or reacting to a Facebook “topic of the day” post) in each Facebook activity.

To describe the general characteristics of engagement activity participants, we used several data sources. RAC members completed a survey as part of the application process in which they self-reported race, ethnicity, gender, income, education, stakeholder type, type of LGG diagnosis, insurance type, and US geographic location. For the #BTSM community, we used Symplur data (which include stakeholder type, eg, patient, clinician, and care partner) and self-reported geographic location for all Twitter accounts that participated in @BTSMchat-hosted tweet chats on March 6, 2022, April 3, 2022, June 5, 2022, and August 7, 2022. Each “live” chat lasted 60 minutes in length, although due to the asynchronous nature of Twitter, participation data include tweets posted using the #BTSM hashtag during the chat and up to 12 hours after each live event; for the first chat only, we also included chats in the 12 hours before the live event. For Facebook group members, the administrator for the Warriors group provided group-level demographics (age, gender, and US-based) generated using Facebook’s Page Admin interface.

### Generation and Analysis of Qualitative Data

We used qualitative content analysis to compare the 3 engagement methods in terms of (1) the nature of the interactions between community members and the research team during engagement activities and (2) recommendations to the LGG Registry. All RAC meeting discussions were audio recorded and then professionally transcribed by a professional transcription service. Twitter transcripts were generated by Symplur, which included all tweet content, the associated Twitter account, time and date of the tweet, and data about participant demographics and stakeholder type (eg, patient, clinician, and care partner). Facebook transcripts were created through screenshot and PDF creation of all comments and reactions to the “topic of the day” posts. All transcripts were uploaded to the qualitative data management software, ATLAS.ti (version 23; ATLAS.ti GmbH).

Qualitative analysis was conducted by a team experienced in qualitative data analysis. Coding and analysis were conducted by 2 data analysts (CR and SGH) and 1 qualitative methodologist (JGB). The analysis was immersive and iterative, beginning with data collection and involving multiple passes through the data to identify deductive and inductive codes to represent the discussion topics (experiences, concerns, and interests of participants). To create the codebook, an inductive approach allowed ideas to emerge [41,42], and deductive codes were added based upon the discussion prompts. Content analysis was completed within and across engagement methods. Preliminary findings were identified for each method, followed by a comparison of the results to identify themes. In detail, rounds of team-based coding were completed by engagement method (in order: RAC, Twitter, and then Facebook) until all 3 engagement methods were fully analyzed across all of their engagement activities. Coders only coded data as linked to a prompt if it were clearly a response to it; data that were not clearly in response to a prompt were coded inductively. Then, the qualitative team compared the preliminary results by engagement method to identify similarities and differences between methods to determine the qualitative results (themes).

The qualitative team met regularly to debrief, refine the codebook, and ensure the codes were applied similarly across coders, thus helping in establishing trustworthiness of the analysis and results [43]. After initial coding calibration, team members double coded 80% of the transcripts to maintain calibration. To further ensure analytic rigor and reliability, the research team engaged in member checking by sharing findings back with participants to see if initial results reflected their experience and capture any missing important discussion points [44]. A reflexive framework guided all aspects of analyses (framing of the analysis, assigning codes, and emerging interpretations of data into themes) [45]. In the final analytic stage, we assessed similarities and differences in the nature of interactions and recommendations for building trust in genomic research and enhancing participation in the LGG Registry among the 3 engagement methods [35,36,38,40,44].

### Engagement Experience Surveys

We chose to evaluate the engagement methods as both a “state” and a “process” [46]. This meant evaluating engagement activities (by having participants fill out surveys after an engagement activity and being specifically told to evaluate that activity) and the overall process of engagement after all 4 activities for each method (in this case, using the Patient Engagement in Research Scale [PEIRS-22]). To do so, at the end of the first and last engagement, participants were invited to complete an online survey evaluating their experience via Qualtrics (Qualtrics International Inc), with a target sample size of 20 survey respondents per method per administration (Multimedia Appendices 3 and 4). Surveys are reported in a manner consistent with the CHERRIES (Checklist for Reporting Results of Internet E-Surveys) checklist for web-based surveys [47] (Multimedia Appendix 5). RAC members received an email invitation via Qualtrics. For Facebook and Twitter, the group administrators posted an announcement about the opportunity to participate in a survey, indicating interested participants should direct message the study lead to receive a link to the survey. Engagement participants also received direct messages from the study lead or group administrator inviting them to complete the survey. Participants received a US $20 gift card for each survey they completed (earning up to US $40 if they completed both surveys).

The postengagement surveys included measures of engagement experience, trust in medical researchers, recollections of which LGG Registry team members were involved, self-reported costs to participate, and demographics. Engagement experience was assessed using 3 established survey measures and some de novo items. The Public and Patient Engagement Evaluation Tool (PPEET) [48] assesses engagement experience, processes, and perceived outcomes of engagement. The 9-item condensed Research Engagement Survey Tool (REST) [49,50] assesses 8 engagement principles based in community-based participatory research, including partner input, capacity building, equity, and trust among partners. The PEIRS [21] is a 22-item survey that has been validated, shortened [51], and even translated into other languages [52,53]. We chose the PEIRS-22 additionally because its items appear capable of evaluating discrete engagement activities (in contrast to the REST, which assesses more long-term, community-based partnerships). PEIRS-22 assesses the overall meaningfulness of engagement on a scale of from 0 to 100 and includes 7 subscales. These subscales include procedural requirements (ie, 7 items assessing team introductions, opportunities to contribute, ability to perform tasks, participation in decisions, receipt of updates, clear communication, and participants’ assessment of time, all on 5-point Likert scales), convenience (ie, 3 items assessing convenience in participating), contributions (ie, 3 items assessing engagement activity and participants’ perceptions of their contributions), team environment and interaction (ie, 2 items assessing perceptions of the team), support (ie, 2 items assessing support to participate), feeling valued (ie, 2 items assessing how participants thought they were valued), and benefits (ie, 3 items assessing how engagement activity participants benefitted from the experience). The 4-item Trust in Medical Researchers Scale was used to assess trust [54].

### Statistical Analysis

Given the low expected sample size for the surveys, these analyses were considered exploratory. Participation data and survey demographic data were analyzed using descriptive statistics (counts, frequencies, and percentages). To our knowledge, no standardized scoring system exists for the PPEET items, which range from “strongly disagree” to “strongly agree” on a 5-point Likert scale; consistent with standard survey practice, these were dichotomized, and the percentage of participants who strongly agreed or agreed with each item was presented. PEIRS-22 subscales were dichotomized using the cutoff values used by the scale’s designers during its validation [51], with respondents with scores indicating low meaningfulness contrasted with those reporting the engagement was moderately, very, or extremely meaningful. For categorical data, tests between groups within each time point were performed using the chi-square test; Fisher exact test was used in cases of low expected cell counts. Continuous survey responses were described using medians and IQRs when the distribution was highly skewed (ie, PEIRS-22 overall scale and REST) and using means and SDs when normally distributed (ie, Trust in Medical Researchers Scale). Comparisons between the 3 engagement methods at each time point were made using Kruskal-Wallis test and 1-way ANOVA. All statistical analyses were performed using SAS (version 9.4; SAS Institute), and *P*<.05 was considered significant.

### Ethical Considerations

This study was approved as exempt human participants research by the Colorado Multiple Institutional Review Board (protocol 20-1001). Participation in engagement activities and analysis of engagement discussion transcripts was not considered human participants research and did not require informed consent. Nonetheless, social media posts included transparency notices, indicating that the activity was being led by a group of researchers and the discussions held may be used to inform research. Only engagement experience surveys were considered human participants research. Survey participants received an information consent before completing the surveys. They were compensated with gift cards worth US $20 for survey completion. All survey data were stored in secure folders accessible only to the research team to protect privacy and confidentiality. Documentation of consent was waived.

## Results

### Engagement Activity Participants

All 25 RAC members participated in at least 1 of the 4 engagement activities reported here (n=24, 96%; n=21, 84%; n=21, 84%; and n=22, 88% participants in RAC meetings 1-4 respectively). There were 197 unique people who participated in tweet chats (n=72; n=37; n=81; n=77 participants in chats 1-4, respectively). Tweet chat participants were a mixture of community representatives, with 45% identified by Symplur, a social media analytics platform, as people with brain tumors (including, but not limited to, LGG). There were 133 unique people who participated in Facebook Warriors page discussions (58, 17, and 88, participants in Facebook post series 1, 2, and 4, respectively [post series #3 excluded; refer to the footnotes in Table 1]). On the basis of Facebook Page Admin statistics, 76% of the group members are aged between 25 and 54 years, 73% identify as women, and 65% live in the United States. Among those people who participated in the engagement activities, there were 92 completed postengagement experience surveys (52 postengagement 1; 40 postengagement 4; refer to Table 2 for survey respondent characteristics). As surveys were anonymous, it is not known how many of the survey respondents were the same across administrations. While not statistically significant due to the small “n” involved, the RAC appeared to have greater representation of those of Black or African American race and Hispanic ethnicity, as well as a more equal self-identified gender balance.

**Table 2:** Characteristics of the engagement survey respondent sample.

			RAC^a^, n (%)				Facebook, n (%)					Twitter, n (%)		
			Post 1 (n=19)		Post 4 (n=17)		Post 1 (n=12)		Post 4 (n=6)		Post 1 (n=21)			Post 4 (n=16^b^)
**Age (** **y)**														
	18-29	4 (21)		3 (18)		0 (0)		0 (0)		2 (10)			2 (13)	
	30-49	9 (47)		9 (53)		9 (75)		4 (67)		18 (86)			8 (50)	
	50-69	6 (32)		5 (29)		3 (25)		2 (33)		1 (5)			6 (38)	
**Gender**														
	Men	10 (53)		8 (47)		1 (8)		1 (17)		5 (24)			7 (44)	
	Women	9 (47)		9 (53)		11 (92)		5 (83)		16 (76)			7 (44)	
	Nonbinary or prefer to self-describe	0 (0)		0 (0)		0 (0)		0 (0)		0 (0)			2 (13)	
**Race**														
	Black or African American	1 (5)		1 (6)		0 (0)		0 (0)		0 (0)			0 (0)	
	Hispanic or Latino ethnicity	2 (11)		3 (18)		0 (0)		1 (17)		0 (0)			0 (0)	
	White	15 (79)		13 (77)		12 (100)		5 (83)		18 (86)			14 (88)	
	Other or unknown^c^	3 (16)		3 (18)		0 (0)		1 (17)		3 (14)			2 (13)	
**Education**														
	High school	1 (5)		1 (6)		1 (8)		1 (17)		2 (10)			1 (6)	
	Some college or associate degree	1 (5)		2 (12)		1 (8)		2 (33)		1 (5)			1 (6)	
	Bachelor’s degree	6 (32)		5 (29)		2 (17)		1 (17)		6 (29)			4 (25)	
	Master’s degree	8 (42)		5 (29)		7 (58)		1 (17)		5 (24)			3 (19)	
	Doctoral or professional degree	3 (16)		4 (24)		1 (8)		1 (17)		7 (33)			7 (44)	
**Household income (US $)**														
	Unknown or prefer not to answer	2 (11)		4 (24)		4 (33)		0 (0)		5 (24)			3 (19)	
	<50,000	5 (26)		4 (24)		2 (17)		2 (33)		2 (10)			0 (0)	
	50,000-99,999	1 (5)		4 (24)		1 (8)		4 (67)		9 (43)			7 (44)	
	≥100,000	11 (58)		5 (29)		5 (42)		0 (0)		5 (24)			6 (38)	
**Participant perspective** ^d^														
	I have personally been diagnosed with a brain tumor.	15 (79)		14 (82)		12 (100)		6 (100)		12 (57)			10 (63)	
	I am a care partner (such as a family member or friend) for someone who has been diagnosed with a brain tumor.	2 (11)		1 (6)		0 (0)		0 (0)		4 (19)			2 (13)	
	I am a researcher who studies brain tumors or topics related to brain tumors.	2 (11)		3 (18)		0 (0)		0 (0)		3 (14)			1 (6)	
	I am a health care provider who cares for people with brain tumors.	2 (11)		1 (6)		0 (0)		0 (0)		3 (14)			4 (25)	
	I am a representative of an advocacy organization or other service organization that addresses issues important to people with brain tumors.	3 (16)		1 (6)		0 (0)		0 (0)		4 (19)			0 (0)	
	I am a community member with a general interest in brain tumors and genomic research.	2 (11)		2 (12)		1 (8)		1 (17)		4 (19)			1 (6)	

^a^RAC: research advisory council.

^b^A total of 17 participants in this group were analyzed for other outcomes, but one chose not to provide any demographic information and was excluded from this table.

^c^Includes participants who identify as Asian (4 respondents), White and Asian (2 respondents), White and Black (2 respondents), American Indian or Alaska Native (1 respondent), and those who did not respond (3 respondents).

^d^Responses may add to >100% as participants were able to select all options that applied.

### Nature of the Engagement Activity Interactions

#### Overview

Qualitative analyses revealed differences in the nature of the interactions among engagement activity participants for each engagement method (Table 3 provides additional illustrative quotes).

**Table 3:** Additional supportive quotations about the differences in the nature of interaction themes by engagement method.

Similar and dissimilar themes			Illustrative quotations from engagement activity participants
**Style of communicating: extent of 2-way communication**			
	Similar—2-way communication	RAC^a^ engagement activity 4: patient, about their views on returning genomic results to patients: “Is he [a research scientist] mostly looking at the genetic material from the tumor itself, or are we also looking at the genetic factors of just the person or the kind of people that tumors occur in for the first place, or where they recur? Then, also, are we also looking at environmental factors or things like that?”; researcher: “Yes, all of those. They are characterizing the tumor, and then they’re doing a whole genome sequencing. Not only can they look at genetic factors for brain tumors, they also will have results for do you have the gene for Huntington’s disease? Do you have the gene for such and such? The results that get returned to individuals could potentially have all of those things in one report.”	
	Dissimilar—posts with limited back-and-forth communication	Facebook engagement activities 3 and 4: in response to a question posted to the Facebook page: “After surgery did you all get a pathology report with your tumor’s IDH mutations and co-deletion status”; patient: “No the dr kept it. Not sure why. He read it to me.” (No other reply or comments posted in response during the Facebook engagement)	
**Sharing of personal challenges with LGG** ^b^			
	Similar—personal challenges with LGG shared	Facebook engagement activity 2: patient: “When I was first diagnosed they didn’t know what I had, the only thing they could tell me was it was slow growing tumor that I’ve had for 10 yrs [years] or more. But then I did what everyone isn’t supposed to do, I went to Google. I put in ‘what do I ask when I see the doctors- oncologist’ and ‘what supplements should I take.’ I found a page that is no longer in use, but that really helped me start the process.” RAC engagement activity 4: patient: “For me, at least there was a roller coaster. Surgery was like, ‘Ah, yes. This was completely successful. I don’t have to worry about this anymore.’ Then a kind of downhill slide of, ‘Oh, I know this is gonna come back.’ Then kind of warring between the two of those, just a mental gymnastics, trying to figure out, ‘Am I going to let myself live my life and trust this is never gonna come back and deal with it if it comes back? Am I going to mentally prepare and kind of live in a semi-state of anxiety?’...Finding that balance has been my biggest hurdle so far.”	
	Dissimilar—direct calls for change in LGG research	Tweet chat engagement activity 4: other advocate: “[researchers should, for] any new drug approved, publish in the Journal of Neuro-Oncology the mutations/pathology report of every GBM/AA/Olglio [sic] patient anonymously etc and how they reacted to that medecine [sic] or combination [of medicines].”	
**Specificity of scientific concepts**			
	Similar—described LGG science and its uncertainties	Tweet chat engagement activity 1: doctor: “I find it hard in #braintumors to be able to explain much about many genetic tests because we still need to learn so much on the implications of a particular mutation in #braintumors vs others.” RAC engagement activity 4: “All I got handed [after surgery] was the 1p19q codeletion with the IDH2 mutation...I was between grade II and grade III. Some oncologists were hesitant about calling it grade II. Some others were hesitant about calling it grade III, because I had seven percent of my tumor cells that were solid grade III. The other 93 percent was grade II...Depending on the doctor or the institution, they would call it either a solid II or a solid III. I asked for it just to be considered a solid III, just out of precaution.”	
	Dissimilar—extent of LGG details differed by engagement method	Facebook engagement activity 1: patient: “I’m so confused on what my low grade glioma actually is. I don’t have the mutations to clearly categorize it.”	

^a^RAC: research advisory council.

^b^LGG: low-grade glioma.

#### Style of Communicating

The groups showed differences in their styles of communicating during engagement activities. Twitter participants and RAC members were more likely than Facebook participants to be involved in 2-way communication with other engagement activity participants, such as through replies to each others’ comments. As an example, a person who identified themselves as a person with LGG, in response to what causes them concern if seen in advertisements about an LGG medical treatment or study, posted the following:

I take it more seriously if it says something about improvements instead. But a cure seems a bit much. I also get suspicious when more than 3 cancers are mentioned. #btsm.

Tweet chat 2

A reply to this tweet posted by a person who identified themselves as a medical provider stated the following:

Instead of promising results, I try to go into the science of why I think a given clinical trial *could* be better than standard of care, based on the available data, but I always, always say that I cannot promise better results, because we’re still figuring that out.

There are multiple examples of a back-and-forth discussion occurring between participants during tweets and RAC engagement activities. This was in contrast to a paucity of back-and-forth exchanges during Facebook engagement activities.

Another similarity in communication style between 2 engagement methods was the sharing of stories about personal challenges. This communication style occurred similarly among members of Facebook and RAC engagement activities and did not occur nearly as often among participants on Twitter. For instance, a Facebook discussion participant with LGG posted the following (Table 3 provides an additional supportive quotation):

When I was first diagnosed they didn’t know what I had, the only thing they could tell me was it was slow growing tumor that I’ve had for 10 yrs or more...I went to Google. I put in “what do I ask when I see the doctors- oncologist” and “what supplements should I take.”

Facebook engagement 2

In comparison, Twitter partners tended to post short and direct calls for change in research process standards for record and specimen acquisition and returning research results to participants with fewer expressions of personal stories. For instance, a person who identified themselves as having LGG posted the following (without sharing any information about their experiences with LGG diagnosis or treatment):

Are there targetable mutations? How might we attempt to stop them? Are there repurposed drugs that could be tried?

Tweet chat 4

#### Specificity of Scientific Concepts

There were qualitative differences across engagement methods in the level of detail and specificity of scientific concepts participants used to express themselves. Twitter and RAC discussions included clinicians and researchers responding directly to questions raised by community members about uncertainty in the current science associated with LGG, the etiology of LGG, or its prognosis:

I find it hard in #braintumors to be able to explain much about many genetic tests because we still need to learn so much on the implications of a particular mutation in #braintumors vs others.

Doctor, tweet chat 1

Similarly, during a RAC engagement, a person with LGG stated the following:

...I had a biopsy for possible recurrence or radiation necrosis...I believe it was the T2-FLAIR that kept growing, that kept getting bigger and bigger...I have the low-grade astrocytoma grade two with the IDH1 mutation, and I would really like to know what the rhyme or reason is [that may cause LGG recurrence].

RAC engagement 4

Conversely, Facebook discussion participants—who tend to be largely people with LGG glioma or care partners, not clinicians or researchers—detailed their desires to better understand their LGG clinical information:

I basically check the internet every day for news on cancer trials/treatments/etc. Of course, it’d be nice to feel like my doctor was doing that for me, especially because she’s better equipped to understand the info & figure out what’s actually pertinent to me.

Patient, Facebook

### Recommendations for Building Trust and Promoting Participation in the LGG Registry

#### Overview

We examined whether the 3 comparator engagement methods would lead to similar recommendations for enhancing trust in genomic research and participation in the LGG Registry. Due to limited volume and details in Facebook comments, recommendations largely reflect input from RAC and tweet chat participants (Table 4). To illustrate, there were 8654 transcribed words in the transcripts across the Facebook engagement activities, whereas the Twitter engagement activities had 35,471 transcribed words and the RAC engagement activities had 127,691 transcribed words. Overall, we found no major qualitative differences in the content of recommendations for the LGG research team generated by each engagement method.

**Table 4:** Additional supportive quotations about trust in genomic research and participation in the Low-Grade Glioma (LGG) Registry.

Trust and participation themes	Illustrative quotations from engagement activity participants
Trust in genomic research and researchers transparently sharing information	RAC^a^ engagement activity 1: patient said the following about the question “Would you want to know before deciding to participate in research that studies LGG genes?”: “I would wanna know, what is the point? What is the end goal? What are we looking for? What do we hope to accomplish with this?...I think a general overview just to know that nothing nefarious is being planned. [With this information,] I think—for the most part-- if folks sign up to participate, there’s an implicit trust implied there.” Tweet chat engagement activity 1: other advocate: “Breaking it [information about research] down and putting it into terms that the patient and care partner could easily understand and grasp would be crucial in my opinion. I think it would also help in strengthening the partnership between patient and researcher.” Facebook engagement activity 2: patient and researcher: “Sharing results in a transparent way is key. As a patient, if you tell me the results aren’t promising, that honesty goes a long way in instilling trust and recruiting participants.”
Support for participation in LGG genomic research and the LGG Registry	RAC engagement activity 1: patient and researcher: “When I got my brain tumor diagnosis—now like 14 years ago—some of like the subpopulations of brain tumors didn’t exist. It was before the reclassification of the different brain tumor stuff. I just decided to share all of my health information because I felt like—to participate in any study because there weren’t any people specifically studying low-grade glioma 14 years ago. That led me to this path to wanna see how I could get involved in as many things as I possibly could, just thinking that the more information is out there, the better...” Tweet chat engagement activity 1: other advocate: “I would want to know how they (researchers) predict the results of this study could help future patients and their care partners.” Facebook engagement activity 1: patient: “I don’t care at all they can have access to every facet of my tumors genetics, my lifestyle, family history, location I grew up, I am more than willing to give up any and all privacy if it helps researchers find a cure for future generations. They can have my brain when I die someday too!”
Data collection and enrollment processes	RAC engagement activity 3: patient: “...if instead of me gathering all the records, I just sign a HIPAA release form...Then you (researchers) go fly off and reach out to those institutions and say, ‘Here’s the HIPAA. We need access to this.’ That’s the easiest thing...Instead of me feeding you the fish, I tell you where the fish is...” Tweet chat engagement activity 3: patient family member and advocate: “When [name] was sick he shuffled between so many institutions, none of whom communicated with each other. It was a nightmare.” Facebook engagement activity 1: patient: “...working with a researcher who is directly introduced to me by my team of doctors really increases my comfortably with everything [sharing specimens and records for research].”
Return of results	RAC engagement activity 3: patient: “...we all are brain-tumor—brain cancer patients. Sometimes we lose a little bit of the understanding of how things work. Having that broke down to where—like I’m a five-year-old is very easy for me to be able to do...I think [that approach] would be very beneficial.” Tweet chat engagement activity 1: patient: “I would want updates on what is being studied and what is being learned, how it is being used. I am a huge research proponent but not if the patients and their care partners get kept in the dark since the research wouldn’t happen without their participation.” Facebook engagement activities 3 and 4: patient said the following about their views returning individual results to patients: “Please let us know what treatments are successful and when they are going to update the survival rate for IDH1 grade 2. There’s IDH1 inhibitors and we need to know if these are working?”

^a^RAC: research advisory council.

#### Trust in Genomic Research and Researchers Transparently Sharing Information

Participants in all 3 engagement groups expressed widespread trust in research and researchers. This trust appeared to be related to positive prior experiences or personal connections with researchers and research institutions. One participant reported the following:

I have worked with researchers. I don’t feel that same sense of detachment from the research environment ‘cause I’m working day in and day out with these folks who I think are outstanding...I don’t feel inclined to have a distrust of the [research] process and I’ve actually participated in some studies and I haven’t had a bad experience.

RAC engagement activity 1

To help build trust in genomic research in general and for participants in the LGG Registry in particular, recommendations included transparent, regular, and clear communication about (1) what is happening with participants’ data and (2) research findings that can help individuals with personal decision-making and improve community health outcomes. Participants emphasized that researchers might build trust by highlighting personal connections with the community and demonstrating affiliation with trusted institutions.

#### Support for Participation in LGG Genomic Research and the LGG Registry

Participants across all 3 engagement methods generally supported LGG Registry participation because it provided an opportunity to help find answers for people with LGG and their families. For instance, 1 participant with LGG emphasized the following:

I don’t care at all they can have access to every facet of my tumors [sic] genetics, my lifestyle, family history, location I grew up, I am more than willing to give up any and all privacy if it helps researchers find a cure for future generations. They can have my brain when I die someday too!

Facebook engagement activity 1

Recommendations for LGG Registry recruitment messages included highlighting the trusted institutions involved, the opportunity to find answers that people with LGG and families care about (such as planning for their futures and about familial risk associated with LGG), and how participant medical records data and genomic information will be kept private and secure. One participant highlighted this last point by recommending a secure place for patients to send their data:

I think also having the repository of where we know we’re sending it. Having assurances on how they’re protecting our privacy as to where we’re sending it to, I think, provides some reassurance to patients if they know that the portal that they’re sending to has certain security in play.

RAC engagement activity 3

#### Data Collection and Enrollment Processes

Participants expressed the need to minimize the burden of obtaining patient health records and specimens; many individuals detailed challenges they had experienced collecting their medical records and specimens. A patient family member and advocate said the following:

When [name] was sick he shuffled between so many institutions, none of whom communicated with each other. It was a nightmare.

Tweet chat 3

Burdens that were described by these participants included both cognitive (eg, remembering or tracking institutional requirements) and physical (eg, traveling to request and receive paper copies and radiography films). Participants emphasized this in part because people with LGG can experience cognitive and other disabilities.

#### Return of Results

If someone agrees to participate in genomic research, they expect to receive their own individual results showing biomarkers and genomics reports and overall research findings in plain language. For instance, 1 person with LGG shared the following:

[W]e all are brain-tumor—brain cancer patients. Sometimes we lose a little bit of the understanding of how things work. Having that [individual genomics results] broke down to where—like I’m a five-year-old...I think [that approach] would be very beneficial.

RAC engagement activity 3

Informing participants of new genomic findings or updates in general for LGG brain tumors was also recommended. While participants want to use individual results for personal and family decision-making, clinicians, researchers, and institutional review board representatives clarified that participants needed to understand that research results might not be validated genomic tests, which they believed are the only results that should inform clinical care or personal or family decisions.

### Engagement Experience Survey Results

Results of the engagement experience surveys (n=52, postengagement 1; n=40, postengagement 4) are shown in Table 5 (PPEET), Table 6 (REST and Trust in Medical Researchers Scale), and Table 7 (PEIRS-22; postengagement 4 only). The PPEET items showed positive ratings of engagement experience overall for RAC participants, where all items were endorsed by at least 70% of respondents; ratings for social media methods were more variable, with the lowest ratings reported by Facebook engagement activity participants, where several items were endorsed by only 50% to 70% of participants. As shown in Table 5, there were statistically significant differences in engagement experience for several PPEET items: belief that participation was diverse (eg, at time point postengagement activity 1: RAC: 100%, Twitter: 91%, and Facebook: 58%; P=.004), belief that a wide range of views were expressed in engagement 1 (RAC: 84%, Twitter: 91%, and Facebook: 50%; *P*=.02), and belief that the activity achieved the stated objectives (RAC: 100%, Twitter: 81%, and Facebook: 67%; *P*=.03).

*P* values obtained from the Kruskal-Wallis test for REST and 1-way ANOVA for TMR. *P* value considered statistically significant at <.05.

**Figure 1:**
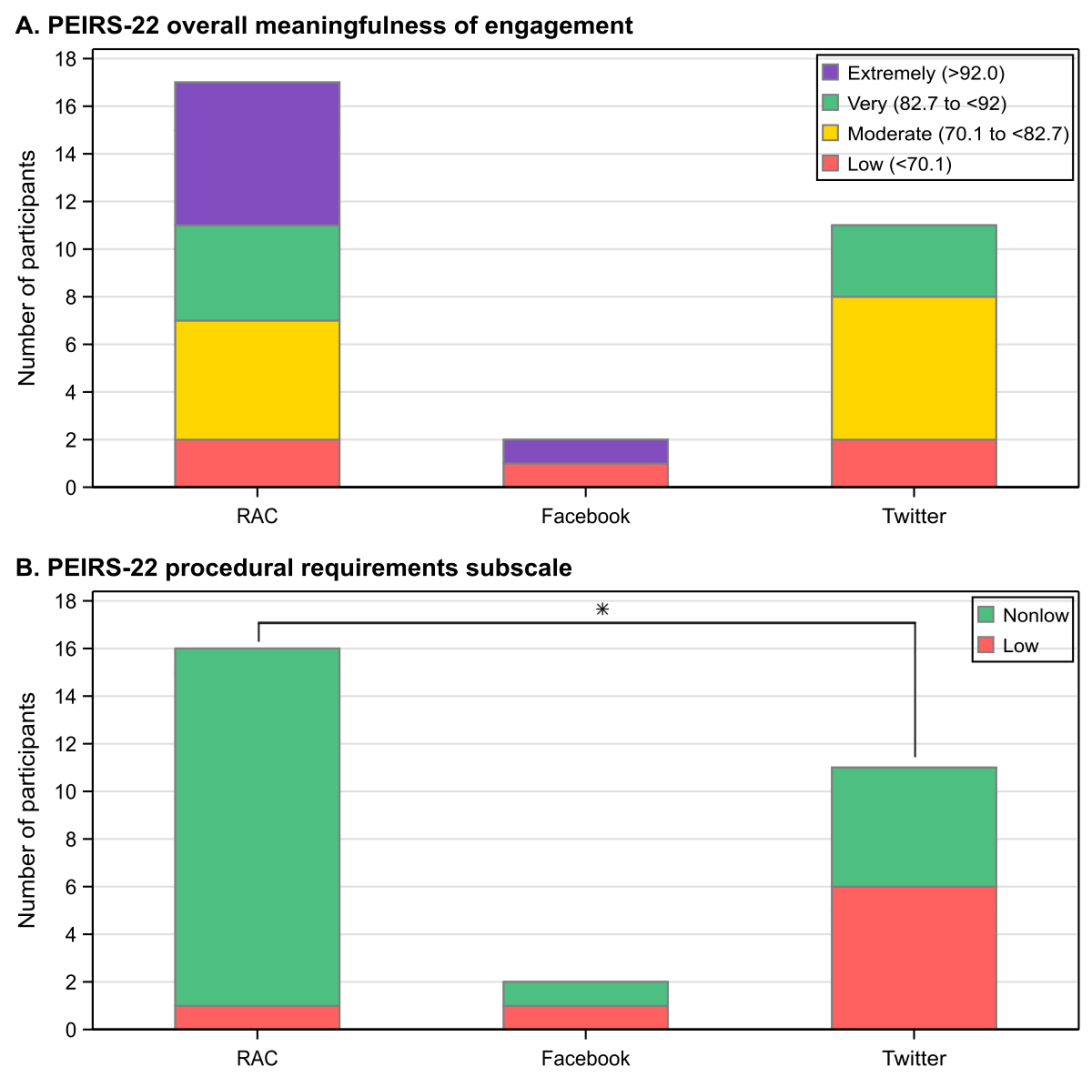
Patient Engagement in Research Scale (PEIRS-22) survey results. RAC: research advisory council.

There were no statistically significant differences between engagement approaches in the overall meaningfulness of engagement, as assessed by the REST or PEIRS-22 (Figure 1A). However, RAC members rated PEIRS-22 procedural requirements more positively (Figure 1B) and reported recalling a wider range of expressed views than social media participants (Table 5); only 6% (1/16) of RAC members assessed the procedural requirements as “low” in terms of meaningfulness, compared to 55% (6/11) of Twitter participants and 50% (1/2) of Facebook participants (*P*=.01).

**Table 5:** Public and Patient Engagement Evaluation Tool (PPEET) engagement experience survey results.

PPEET item	Research advisory council, n (%) (agree/strongly agree)		Facebook, n (%) (agree/strongly agree)		Twitter, n (%) (agree/strongly agree)			Group comparison *P* values^a^	
	Post 1 (n=19)	Post 4 (n=17)	Post 1 (n=12)	Post 4 (n=6)	Post 1 (n=21)	Post 4 (n=17)	Post 1		Post 4
Overall, I was satisfied with this activity	18 (95)	15 (88)	11 (92)	5 (83)	21 (100)	17 (100)	.51		.36
The purpose of the activity was clearly explained	18 (95)	16 (94)	11 (92)	5 (83)	20 (95)	16 (94)	>.99		.53
I had enough information to contribute to the topic being discussed	19 (100)	15 (88)	12 (100)	5 (83)	19 (91)	16 (94)	.34		.80
I feel that my views were heard	19 (100)	15 (88)	11 (92)	5 (83)	19 (91)	17 (100)	.44		.36
This activity was a good use of my time	19 (100)^b^	14 (82)	10 (83)	5 (83)	21 (100)	17 (100)	.05		.19
I was able to express my views freely	19 (100)	16 (88)	10 (83)	5 (83)	20 (95)	17 (100)	.23		.36
I feel that the input provided through this activity will be considered by the organizers	19 (100)	17 (100)	12 (100)	4 (67)	18 (86)	16 (94)	.17		.05
This activity included diverse participants from different backgrounds and walks of life	19 (100)	12 (71)	7 (58)	3 (50)	19 (91)	16 (94)	.004		.05
A wide range of views on the topic were expressed	16 (84)	14 (82)	6 (50)	5 (83)	19 (91)	17 (100)	.02		.19
The activity achieved its stated objectives	19 (100)	12 (71)	8 (67)	4 (67)	17 (81)	16 (94)	.03		.12
The supports I needed to participate were available (eg, travel, child care, technology)	16 (84)	17 (94)	8 (67)	4 (67)	15 (71)	16 (94)	.50		.24
As a result of my participation in this activity, I have greater trust in the researchers who are leading the Low Grade Glioma Registry	18 (95)	15 (88)	9 (75)	3 (50)	14 (67)	13 (77)	.08		.14
I think this activity will make a difference	18 (95)	13 (77)	9 (75)	4 (67)	16 (76)	10 (59)	.23		.55
I understand how the input from this activity will be used.	15 (79)	14 (82)	10 (83)	4 (67)	16 (76)	11 (65)	>.99		.56
As a result of my participation in this activity, I am better informed about the Low Grade Glioma Registry	16 (84)	14 (82)	8 (67)	4 (67)	14 (67)	11 (65)	.39		.56

^a^*P* values obtained from chi-squared tests or Fisher exact test. *P* value considered statistically significant at <.05.

**Table 6:** Research Engagement Survey Tool (REST) and Trust in Medical Researchers (TMR) engagement experience survey results. REST: mean score of items scored 1 to 5, poor to excellent. TMR: scored 1 to 5, strongly disagree to strongly agree, with negative items reverse-coded so that higher score indicates more trust. Theoretical range was 4 to 20.

	RAC^a^ post 1 (n=19), median (IQR)	RAC post 4 (n=17), median (IQR)	Facebook post 1 (n=12^b^), median (IQR)	Facebook post 4 (n=6), median (IQR)	Twitter post 1 (n=21), median (IQR)	Twitter post 4 (n=17), median (IQR)	Post 1, *P* value	Post 4, *P* value
REST	4.1 (4.0-4.6)	4.0 (3.6-4.3)	3.9 (3.2-4.8)	3.3 (3.0-4.7)	4.3 (3.3-4.5)	4.2 (3.8-4.5)	.71	.33
TMR	14.2 (3.3)	13.6 (2.6)	14.8 (2.5)	13.2 (2.8)	13.6 (2.0)	14.9 (2.4)	.48	.24

^a^RAC: research advisory council.

^b^A total of 12 participants had values for TMR and 11 participants had values for REST.

**Table 7:** Postengagement 4 Patient Engagement in Research Scale (PEIRS-22) responses overall and by domain across engagement methods.

			RAC^a^ post 4 (n=17)		Facebook post 4 (n=6)		Twitter post 4 (n=16)		Differences among groups, *P* value^b^	
PEIRS-22 (range 0-100), median (IQR)			87.5 (75.0-94.3)		78.9 (59.1-98.7)		77.3 (71.2-85.2)		.24	
**PEIRS degree of meaningfulness**										.12
	Low (<70.1), n (%)	2 (12)		1 (50)		2 (18)				
	Moderately (70.1 to <82.7), n (%)	5 (29)		0 (0)		6 (55)				
	Very (82.7 to <92.0), n (%)	4 (24)		0 (0)		3 (27)				
	Extremely (>92.0-100), n (%)	6 (35)		1 (50)		0 (0)				
**PEIRS degree of meaningfulness**										.40
	Low (<70.1), n (%)	2 (12)		1 (50)		2 (18)				
	Moderately, very, and extremely (≥70.1), n (%)	15 (88)		1 (50)		9 (82)				
	Missing, n	0		4		5				
**Procedural requirements (range 0-31.8)**										.01
	Low (<22.3), n (%)	1 (6)		1 (50)		6 (55)				
	Moderately, very, and extremely (≥22.3), n (%)	15 (94)		1 (50)		5 (46)				
	Missing, n	1		4		5				
**Convenience (range 0-13.6)**										.23
	Low (<9.6), n (%)	3 (18)		3 (50)		2 (13)				
	Moderately, very, and extremely (≥9.6), n (%)	14 (82)		3 (50)		13 (87)				
	Missing, n	0		0		1				
**Contributions (range 0-13.6)**										.14
	Low (<9.6), n (%)	1 (6)		2 (40)		2 (13)				
	Moderately, very, and extremely (≥9.6), n (%)	16 (94)		3 (60)		14 (88)				
	Missing, n	0		1		0				
**Team environment and interaction (range 0-9.1)**										.72
	Low (<6.4), n (%)	4 (24)		0 (0)		3 (23)				
	Moderately, very, and extremely (≥6.4), n (%)	13 (77)		4 (100)		10 (77)				
	Missing, n	0		2		2				
**Support (range 0-9.1)**										.67
	Low (<6.4), n (%)	1 (6)		0 (0)		2 (18)				
	Moderately, very, and extremely (≥6.4), n (%)	16 (94)		3 (100)		9 (82)				
	Missing, n	0		3		5				
**Feel valued (range 0-9.1)**										>.99
	Low (<6.4), n (%)	2 (13)		0 (0)		1 (8)				
	Moderately, very, and extremely (≥6.4), n (%)	14 (88)		3 (100)		12 (92)				
	Missing, n	1		3		3				
**Benefits (range 0-13.6)**										.27
	Low (<9.6), n (%)	1 (6)		1 (33)		2 (13)				
	Moderately, very, and extremely (≥9.6), n (%)	16 (94)		2 (67)		13 (87)				
	Missing, n	0		3		1				

^a^RAC: research advisory council.

^b^*P* values obtained from the Kruskal-Wallis test for the continuous overall scale. *P* values obtained from chi-square tests or Fisher exact test for categorical comparisons. *P* value considered statistically significant at <.05.

## Discussion

### Principal Findings

This study was designed to compare advisory council versus social media–based methods for engaging patients and other community representatives in the planning and conduct of LGG genomic research. To our knowledge, this was the first study designed prospectively to evaluate differences between advisory council and social media–based research engagement methods. Qualitative analyses revealed minimal content differences in the insights generated by each engagement method; recommendations for how the OPTIMUM project may improve trust, promote participation in LGG genomic research, manage data collection, and return individual and research results were similar across engagement methods. However, the 3 methods exhibited differences in the number and types of people engaged, the nature of interactions between researchers and engagement activity participants, and how participants experienced the engagement process.

We observed differences in the PEIRS-22 procedural subscale between the 3 engagement methods, with the RAC rating engagement procedures more favorably; no differences were found in the other subscales. For the other subscales, this may be because the participating members of the research team were consistent across engagement methods (meaning differences in the team and environment were mitigated), all methods were considered convenient for individual participants (who volunteered to participate), and so on. For the procedural subscale, the fact that a RAC comes with an expectation of a standing advisory committee over time could mean that participants are more likely to feel introduced to the research team, have opportunities to contribute, and participate in decisions—all core elements of that subscale.

Importantly, we do not conclude that a RAC is inherently “better” than other methods; instead, we aim to highlight relevant differences. A RAC may provide consistent engagement with the same individuals, a relatively simple structure, and the ability to purposively select members for diversity. However, a RAC may not have the reach of social media engagement, which can engage more diverse individuals, and social media engagement may be less labor or resource intensive for learning important insights (as our study found similar qualitative themes). However, our findings suggest this is not a given; social media did not necessarily yield more diverse participants, and additional efforts may be needed to promote diversity and inclusivity for those methods of engagement. These relevant differences inform selecting the type of engagement activity based on the research question and population. For instance, a research topic that requires a higher level of scientific understanding may best plan to engage Twitter and RAC communities, as the quantity and quality of Facebook data may be limited. Likewise, Facebook and RAC engagement may better suit a research question best understood through narrative interaction between participants, as these engagements were where we observed participants organically responding to one another.

As expected, the advisory council method represented fewer total individuals than those engaged through social media. We had aimed to broaden both total numbers and the representativeness of those participating by using social media methods. Contrary to expectations, our results show that the RAC was more diverse in terms of race and ethnicity than those engaged through social media. The OPTIMUM project team translated findings from this study into several types of decisions. First, the results informed how we would continue to engage the LGG community throughout the remaining conduct and dissemination of the research, with both RAC and social media engagement continuing throughout the project period, although the RAC meets more regularly and is more involved in all aspects of study conduct and dissemination. Second, the results informed content for recruitment messages and channels (eg, an emphasis on recruitment via clinical settings rather than through online channels), content for the LGG Registry website and social media pages, and decisions to allocate more project resources to partnerships with additional clinical sites. The need for mitigating data and specimen collection burden—especially regarding access to electronic health records, pathology reports, and tumor samples—was a key insight.

Our study adds to the literature on the science of engagement in important ways. Although research shows that engagement can influence which questions are asked, how studies are conducted, and how findings are shared with relevant communities [1-12], few studies examine which specific engagement methods are most effective for particular purposes or populations. Prior studies have examined the effect of panel composition (eg, Delphi panels [55]) and compared the experience of patients versus researchers within a single method [26]. One previous study used surveys to examine online versus in-person focus group engagement regarding rural health care (not research); it found greater satisfaction among in-person participants and a lack of representativeness [27]. Others compared online voting, in-person focus groups, and mailed surveys in a low back pain data registry; using a qualitative evaluation, results showed that all methods generated similar research priorities but a better experience among in-person focus groups [56].

Unlike these previous studies, we used both qualitative and quantitative data (validated surveys and scales) prospectively to compare the 3 methods of engagement. Similar to these previous studies, we found few differences in the content of recommendations, but we did find differences in communication styles and how recommendations were expressed. Importantly, we found few quantitative differences in participants’ overall assessment of engagement methods or their overall trust in research. Together, these findings suggest that all 3 methods may have a place in OPTIMUM LGG genomic research engagement. Experience may be optimized when participants are able to choose methods with which they are comfortable.

Our results have implications for how to engage community in research more effectively and hypotheses for future research. Participants in the advisory council rated the procedural elements of engagement (eg, proper introductions, opportunities to contribute, bilateral communication, and whether the activity is worth one’s time) more highly. If social media engagement included more informal or unstructured time, these findings might change. Unexpected results showed that our RAC members were less likely to report feeling their views were heard. This finding could reflect the opinions of council members who felt uncomfortable speaking, the existence of more outgoing and outspoken personalities in a group, or different expectations among social media participants about what it means to feel heard. Third, despite the arguably broader reach of social media, advisory council participants perceived greater diversity of views. This could reflect the intentional recruitment efforts that RACs often involve, as ours did, or the persistence of a digital divide.

As a pilot exploratory study of the comparative effectiveness of engagement methods, we interpret our findings cautiously but can still offer several recommendations for researchers. First, our study demonstrates the feasibility of conducting an evaluation of engagement processes alongside engagement activities and the ability to detect differences even with relatively small numbers. Related to this, we found the engagement survey tools and items we used to be appropriate for these methods and this context, although adapted tools and items may be needed for other communities. Second, although qualitatively we found no differences in the content of recommendations among groups, we do not suggest they are therefore equal. Issues of cost, time, and expertise, for example, are likely to matter; a researcher already familiar with advisory panel methods is likely to do a better job with that method compared to social media formats, for example, and not all communities have active online communities.

Third, our findings suggest that the presumed advantages and disadvantages of each method are not fixed or guaranteed. Engagement via online social media sites did not inherently appear to increase reach or diversity; researchers may need to take extra steps to identify and recruit for diversity or use other methods to fill these gaps. Likewise, an advisory panel—despite engaging a small number of individuals—did not necessarily mean all voices were heard; a researcher will still need to use techniques within meetings (eg, use of a talking object that is passed around a real or virtual room) to allow all individuals an opportunity to contribute. Finally, a robust, comprehensive, and comparative assessment of engagement methods in different contexts and for different purposes is still an unmet need, particularly for underrepresented research groups and groups considered marginalized. Further research is needed to improve and validate engagement measures used (eg, in languages other than English). Patient partners (coauthors of this paper) emphasize that the results of this study highlight opportunities to engage the brain tumor community not only in genomic research but also in a wide range of other priority areas, such as quality of life research.

### Limitations

The principal limitation of this study was that this analysis was secondary to the primary purpose of the engagement activities—which was to establish bidirectional communication and build relationships with the LGG community. The fact that we used existing engagement structures via the RAC, Twitter, and Facebook meant that participants self-selected into engagement methods that may have matched their preferred style of communication. This approach is respectful of engagement principles; that we observed statistically significant differences and meaningful qualitative differences between groups is thus still important, and future research should explore the possibility and permissibility of randomized designs. Moreover, although we sought to evaluate the engagement state after specific activities in engagements 1 and 4 as well as the process from beginning to end using the PEIRS-22, the wording of items and participants’ own perceptions of them can be subject to interpretation. This too requires further research.

In addition, the data used for qualitative analysis were not gathered using standard qualitative research methods, such that the usual ability to follow up with a respondent to ask for clarification or more information about a statement was not present. The high degree of trust in medical research reported in postengagement surveys suggested that we failed to engage community members with less trust in research. The resulting recruitment messages may therefore underemphasize key points needed to enhance trust. For instance, those engaged suggested that noting the involvement of respected research institutions and naming scientists involved would enhance trust in the LGG Registry, but that might not be the opinion of those without previous good experiences with academic institutions. Although we partnered with the community in engaging Facebook participants, other strategies—different Facebook groups, different social media platforms, or nonsocial media–based engagement—may be required to gather more diverse perspectives.

### Conclusions

Engagement of patients, families, and other community partners in research is an ethical imperative. To do so with authenticity requires that we evaluate engagement methods for their effectiveness across diverse contexts, and we must hold our engagement methods to the same rigorous standard of evidence as our research methods. Our study demonstrates the feasibility of comparative evaluation of engagement methods that can further inform engagement approaches. Future research should examine additional methods comparatively in different research settings and communities and for different purposes.

## Appendix

Multimedia Appendix 1 Research advisory council descriptive characteristics.

Multimedia Appendix 2 Engagement discussion prompts.

Multimedia Appendix 3 Postengagement survey 1.

Multimedia Appendix 4 Postengagement survey 4.

Multimedia Appendix 5 CHERRIES (Checklist for Reporting Results of Internet E-Surveys) checklist.

## Abbreviations

#BTSM: brain tumor social media
CHERRIES: Checklist for Reporting Results of Internet E-Surveys
LGG: low-grade glioma
NCI: National Cancer Institute
PE-CGS: Participant Engagement and Cancer Genome Sequencing
PEIRS: Patient Engagement in Research Scale
PPEET: Public and Patient Engagement Evaluation Tool
RAC: research advisory council
REST: Research Engagement Survey Tool

## Data Availability

The datasets generated or analyzed during this study are not publicly available due to identifiability concerns but are available from the corresponding author on reasonable request.
