# Supplementary material for: Engagement Methods in Brain Tumor Genomic Research: Multimethod Comparative Study
Source: J Particip Med. 2025 Aug 21;17:e68852. doi: 10.2196/68852 (PMC12411796; doi:10.2196/68852)
Supplement: Multimedia Appendix 1 [file jopm_v17i1e68852_app1.docx]

**A Multi-Method Comparative Study of Engagement Methods in Brain Tumor Genomic Research**

Matthew DeCamp, MD, PhD;^1,2^ Juliana G. Barnard, MA;^3,4^ Carly Ritger, MS;^3^ Laura J. Helmkamp, MS;^3^ Anowara Begum, MPH;^3^ Sandra Garcia-Hernandez, MPH;^3^ Rudy Fischmann;^5^ Nestelynn Gay, MS;^5^ Ricardo Gonzalez-Fisher, MD, MPH;^5^ Kevin Johnson, PhD;^5,6^ Lindsay A. Lennox, BA;^7^ Guy R. Lipof;^5^ Jasmyn Ostmeyer, M.Ed;^5^ Ifeoma Perkins, MD,^2,8^ Laura Pyle, PhD;^4^ Liz Salmi, AS;^9^ Talia Thompson, PhD;^3,4^ Elizabeth B. Claus, MD, PhD;^10,11^ Roel Verhaak, PhD;^6,12^ Bethany M. Kwan, PhD^3,7^

**Affiliations**

^1^ Division of General Internal Medicine, University of Colorado School of Medicine, Aurora, CO

^2^ Center for Bioethics & Humanities, University of Colorado School of Medicine, Aurora, CO

^3^ Adult & Child Center for Outcomes Research & Delivery Science, University of Colorado Anschutz Medical Campus, Aurora, CO

^4^ Department of Pediatrics, University of Colorado School of Medicine, Aurora, CO

^5^ Low Grade Glioma Registry Research Advisory Council

^6^ Department of Neurosurgery, Yale School of Medicine, Yale University, New Haven, CT, USA

^7^ Department of Emergency Medicine, University of Colorado School of Medicine, Aurora, CO

^8^ Department of Pathology, University of Colorado School of Medicine, Aurora, CO

^9^ Department of Medicine, Beth Israel Deaconess Medical Center, Boston, MA

^9^ Children’s Hospital Colorado

^10^ Yale School of Public Health, New Haven, CT

^11^ Department of Neurosurgery, Brigham and Women’s Hospital, Boston, MA

^12^ Department of Neurosurgery, Amsterdam University Medical Center, Amsterdam, The Netherlands

The Patient – Patient-Centered Outcomes Research

**Corresponding author**: Bethany M. Kwan, PhD, MSPH; [bethany.kwan@cuanschutz.edu](mailto:bethany.kwan@cuanschutz.edu)

**Supplemental Table 1. LGG Registry RAC Member Characteristics**

| **Stakeholder Role (n=25) (not mutually exclusive)** | **N (%)** |
| --- | --- |
| Person living with LGG | 19 (76%) |
| Patient community leader | 3 (12%) |
| Regulatory Expert | 2 (8%) |
| Health Care Provider for people with LGG | 1 (4%) |
| Genetics expert | 1 (4%) |
| Advocacy organization representative | 1 (4%) |
| Care Partner | 1 (4%) |
| **Gender (n=25)** |  |
| Woman | 13 (52%) |
| Man | 12 (48%) |
| **Age (n=25)** |  |
| 18-24 years old | 2 (8%) |
| 25-34 years old | 8 (32%) |
| 35-44 years old | 5 (20%) |
| 45-54 years old | 9 (36%) |
| 55-64 years old | 1 (4%) |
| **Highest level of education (n=25)** |  |
| Some college but no degree | 3 (12%) |
| High school graduate (high school diploma or equivalent including GED) | 1 (4%) |
| Associate degree in college (2-year) | 1 (4%) |
| Bachelor's degree in college (4-year) | 6 (24%) |
| Master's degree | 9 (36%) |
| Doctoral degree | 1 (4%) |
| Professional degree (JD, MD) | 4 (16%) |
| **Hispanic/Latino ethnicity (n=25)** |  |
| Yes | 4 (16%) |
| No | 21 (84%) |
| **Race (n=25) (select all that apply)** |  |
| White | 21 (84%) |
| Black or African American | 3 (12%) |
| Asian | 1 (4%) |
| Other | 1 (4%) - (Middle Eastern) |
| **LGG diagnosis (n=25)** |  |
| Astrocytoma | 8 (32%) |
| Oligodendroglioma | 8 (32%) |
| Other low grade glioma | 3 (12%) |
| No, I have not personally been diagnosed with a low grade glioma | 6 (24%) |
| **Insurance (n=25)** |  |
| Private insurance provided by my or a partner or parent's employer | 20 (80%) |
| Private insurance purchased on my own (e.g., through HealthCare.gov or a state insurance marketplace) | 2 (8%) |
| Other type of insurance | 3 (12%) - Private by Employer and Medicare |
| **Income (n=25)** |  |
| Less than $50,000 | 5 (20%) |
| $50,000 to $99,999 | 4 (16%) |
| $150,000 or more | 7 (28%) |
| I prefer not to say | 9 (36%) |
