# Supplementary material for: Engagement Methods in Brain Tumor Genomic Research: Multimethod Comparative Study
Source: J Particip Med. 2025 Aug 21;17:e68852. doi: 10.2196/68852 (PMC12411796; doi:10.2196/68852)
Supplement: Multimedia Appendix 2 [file jopm_v17i1e68852_app2.docx]

**A Multi-Method Comparative Study of Engagement Methods in Brain Tumor Genomic Research**

Matthew DeCamp, MD, PhD;^1,2^ Juliana G. Barnard, MA;^3,4^ Carly Ritger, MS;^3^ Laura J. Helmkamp, MS;^3^ Anowara Begum, MPH;^3^ Sandra Garcia-Hernandez, MPH;^3^ Rudy Fischmann;^5^ Nestelynn Gay, MS;^5^ Ricardo Gonzalez-Fisher, MD, MPH;^5^ Kevin Johnson, PhD;^5,6^ Lindsay A. Lennox, BA;^7^ Guy R. Lipof;^5^ Jasmyn Ostmeyer, M.Ed;^5^ Ifeoma Perkins, MD,^2,8^ Laura Pyle, PhD;^4^ Liz Salmi, AS;^9^ Talia Thompson, PhD;^3,4^ Elizabeth B. Claus, MD, PhD;^10,11^ Roel Verhaak, PhD;^6,12^ Bethany M. Kwan, PhD^3,7^

**Affiliations**

^1^ Division of General Internal Medicine, University of Colorado School of Medicine, Aurora, CO

^2^ Center for Bioethics & Humanities, University of Colorado School of Medicine, Aurora, CO

^3^ Adult & Child Center for Outcomes Research & Delivery Science, University of Colorado Anschutz Medical Campus, Aurora, CO

^4^ Department of Pediatrics, University of Colorado School of Medicine, Aurora, CO

^5^ Low Grade Glioma Registry Research Advisory Council

^6^ Department of Neurosurgery, Yale School of Medicine, Yale University, New Haven, CT, USA

^7^ Department of Emergency Medicine, University of Colorado School of Medicine, Aurora, CO

^8^ Department of Pathology, University of Colorado School of Medicine, Aurora, CO

^9^ Department of Medicine, Beth Israel Deaconess Medical Center, Boston, MA

^9^ Children’s Hospital Colorado

^10^ Yale School of Public Health, New Haven, CT

^11^ Department of Neurosurgery, Brigham and Women’s Hospital, Boston, MA

^12^ Department of Neurosurgery, Amsterdam University Medical Center, Amsterdam, The Netherlands

The Patient – Patient-Centered Outcomes Research

**Corresponding author**: Bethany M. Kwan, PhD, MSPH; [bethany.kwan@cuanschutz.edu](mailto:bethany.kwan@cuanschutz.edu)

| **Topic** | **Discussion Prompts** | | |
| --- | --- | --- | --- |
|  | **RAC** | **Twitter/X** | **Facebook LGG group** |
| Trust and benefits of genomic research | *Dates: 3/28/2022, 3/29/2022*  T1: How might research into low grade glioma tumor genes help you as a… - brain tumor patient? - care partner? - community advocate? - clinician? - scientist? - other stakeholder?  T2: For Patients/Care Partners:  - How comfortable are you w/sharing information about your/your loved one’s low grade glioma tumor genes for research?  - How would you feel if it were possible to identify your/your loved ones as a result of research?T2: For Clinicians/Researchers: - What worries about information privacy have you observed among patients/care partners when you discuss brain tumor genes?😟 - What impacts do you consider when answering questions about genetic + genomic test results?  T3: Low grade glioma genomic research provides information about how tumors change over time, and may be used to identify treatments.  What would you want to know BEFORE you decided to participate in research that studies low grade glioma genes?  T4: Sending medical records + tumor samples can feel like a black box.   Patients/Care Partners: What would you expect researchers to tell you about what’s happening with your data and tumor samples? Clinicians: What updates do you receive about your patients? | *Date: 3/6/2022*  T1: How might research into brain tumor genes 🧠🧬🎯 help you as a… - brain tumor patient? - care partner? - community advocate? - clinician? - scientist? - other? #BTSM  T2: For Patients/Care Partners:  - How comfortable are you w/sharing information about your/your loved one’s brain tumor genes for research?🔬  - How would you feel if it were possible to identify your/your loved ones as a result of research?🕶️ #BTSM. T2: For Clinicians/Researchers: - What worries about information privacy have you observed among patients/care partners when you discuss brain tumor genes?😟 - What impacts do you consider when answering questions about genetic + genomic test results? #BTSM  T3: Brain tumor genomics provide information about tumors and outcomes, and may be used to identify treatments.🧠🧬🔬💊🎯  What would you want to know BEFORE you decided to participate in research that studies brain tumor genes? #BTSM  T4: Sending medical records + tumor samples can feel like a black box.📤⁉️  Patients/Care Partners: What would you expect researchers to tell you about what’s happening with your data and tumor samples?🗣️ Clinicians: What updates do you receive about your patients? #BTSM | *Dates: 5/15/2022 – 5/20/2022*  Topic of the day #1: How might research into the genetic makeup of low grade glioma tumors help you?  Did you know? Brain tumor genomics (the study of brain tumor genetic makeup) may provide information about tumors and outcomes. Understanding brain tumor genes may be used to identify which treatments will work better for different people.  Want to learn more? Watch this video from the National Brain Tumor Society: <https://www.youtube.com/watch?v=IfkI8QYrzYQ&t=36s>  Topic of the day #2: What might help you feel comfortable sharing information about your or your loved one’s low grade glioma tumor’s genes for research?  How would you feel if it were possible to identify you or your loved one as a result of research?  Topic of the day #3: What would you want to know BEFORE you decided to participate in research that studies brain tumor genes?  Low grade glioma tumor genomics provide information about tumors and outcomes and may be used to identify treatments.  Topic of the day #4: What would you expect researchers to tell you about what’s happening with your data and tumor samples?  Participating in low grade glioma genomic research means sending copies of medical records and tumor samples. This can feel like a “black box.” |
| Registry recruitment messages, materials, protocols | *Dates: 4/25/2022, 4/26/2022*  T1: Information sources: What kinds of messaging have you seen or heard offering information about low grade glioma (or brain cancer in general) treatments, research studies, or general advice? Where have you seen or heard these messages? [Note: this can be about information they actively seek out or that they happen upon.]  For clinicians, reframe the question around “Do your patients ever ask you about information/messages they get about treatments or research opportunities? What kinds of messages/information do they get and from where?  For researchers: How and where do you recruit for research opportunities? What kinds of messages tend to work well?  For IRB: What are the “rules” around messaging and recruitment for research? What’s allowed, and how do you decide to approve it?  T2: Deciding what is “good” information: What are some examples of treatment, research, or general advice that was helpful? How do you decide what messages and information about low grade glioma/brain cancer treatments, research studies, or general advice are worth paying attention to?  For clinicians: How do you help your patients figure out what’s “good” information?  For researchers/IRB: What’s your advice about the best way to convey that a research opportunity is trustworthy and worthwhile, without overpromising?  T3: Deciding what is “not as good” information: What are some examples of treatment, research, or general advice that was not helpful or turned out poorly? How do you figure out if the information may not be trustworthy? What are your personal red flags (or alarm bells) that a low grade glioma/brain cancer treatment, research study, or general advice is “too good to be true”?  For clinicians: What are some red flags you advise your patients to look for?  For researchers/IRB: What are some “no no’s” in messaging about research? How do you make sure that your recruitment messages don’t overpromise or mislead people about what they will get out of the research or the benefits to them?  T4: Feelings about “personalized” or “targeted” information: As far as you know, have you ever received targeted messages or promotions by hospitals, drug companies, or researchers based on your personal information (e.g., a diagnosis, where you live, related internet searches)?  If so, what has been your response to this kind of personalized messaging?  For clinicians/researchers/IRB: What do you think about personalized or targeted messaging about health care or research opportunities? | *Date: 4/3/2022*  T1. Do you see products, services, or trials advertised to people facing brain tumors? If, so where?  How do you decide if what is being promoted is trustworthy or not? #BTSM  #BTSM  T2: Have you ever been targeted by hospitals, drug companies, or researchers based on your personal information?  If so… how does this kind of online targeting (or personalization) make you feel? #BTSM  T3: 🚩🚩🚩 What are your personal red flags that a medical treatment/cure/study is “too good to be true”? #BTSM  T4 for Patients/Care Partners:  What are some good examples of brain tumor tips/treatments/trials that have helped you/your loved one?  What turned out to be a negative experience? #BTSM  T4 for Clinicians/Researchers:  What are some good examples of brain tumor tips/treatments/trials you share with patients/their loved ones? In hindsight, what turned out to be not helpful? #BTSM | *Dates: 6/24/2022 – 6/27/2022*  Information Overload Topic of the Day #1: I notice this group is so great about sharing information with each other. Answering questions, giving tips and honest advice - even when it's a tough message to hear.  Where do you all get your best information to share  with the group? Share a link if you can!  Information Overload Topic of the Day #2: Treatments, nutrition, understanding reports, research studies, advice about living with a brain tumor...... Do you have a way to separate what's helpful information from what's “too good to be true”? Any red flags to look out for?  Information Overload Topic of the Day #3: As far as you know, have you ever received targeted emails, phone calls, or ads from hospitals, drug companies, or researchers based on your personal information (e.g., a diagnosis, where you live, related internet searches)? How do you feel about this? Creepy or cool?  Information Overload Topic of the Day #4: When my mom was sick with a high grade glioma in 2016, she was only offered a few chances to be part of research. As a researcher myself, I wanted to make it easier for people with brain tumors to be part of research. Can you help?  How do you think researchers should share information about how to join a research study? What's off limits? |
| Message engagement testing – Facebook only | n/a | n/a | *Dates: 7/5/2022 – 8/12/2022. Note: to increase engagement with discussion prompts in the LGG Warriors group, multiple message format were tested during this time frame. Engagement with the following set of posts was used to inform future engagement with this group, but was not analyzed as part of this study.*  Post/question: Has anyone heard of All of Us? One of my docs has encouraged me to participate and it makes me nervous. Apparently you can opt to have your “results” returned to you and I know myself well enough to know I wouldn’t be able to resist…#NotSureIWantToKnow  Post/link: The National Brain Tumor Society has a special set of funds just for doing research on oligos. In 2020, they posted this nice report on all the projects, in case you're interested. <https://braintumor.org/.../2020_Oligo_Progress_Report.pdf>  Post/image: “I'm fascinated by my hair loss pattern based on the radiation site (for me: left parietal lobe). I'm four weeks into radiation and I have two weeks left. As a 42yo with no other health conditions, I'm feeling good so far!  Poll: I'm curious if anyone has ever been part of a research study. My mom did a clinical trial for her glioma once the standard surgery+temozolomide+radiation protocol was done.  Post/question: How are you feeling on glioblastoma day?  Poll: What would you do if your oligo/astro came back? Have you talked with your doctor about recurrence? What options do you have left?  Meme: 3 out of 4 of you said you'd been part of research #graymatters [image: ‘galaxy meme’ with text “Let’s Make Brain Cancer Research Shine Bright”]  Meme: What if I told you there was a 3rd button and it was called “patient co-designed research?” Would you click on that button? [image: meme showing 2 buttons labeled “watch and wait” and “the same 3 therapies for the last 40 years”]  Meme: I’ll just leave it here… [image: what gives people feelings of power] |
| Data collection messages, materials and protocols | *Dates: 5/16/2022, 5/19/2022*  T1: Getting data from here to there: What has been your experience with trying to access digital health data – for your own care, for that of a family member, for research, for clinical care?  Think about the last time you tried to get access to health data (broadly speaking - from electronic health records or health “apps” or tools like a FitBit). What was that process like? How did it go?  T2: The patient as data gatherer: If you were asked to gather all “digital health data” for low grade glioma research, where would you start?  What challenges would you anticipate, or have you encountered, from your perspective as a patient, care partner, researcher, clinician?  T3: Support for LGG Registry data gathering:  For patients/care partner:  What might make it easier to share digital health data for LGG research?  What education or information would you need to figure out how to share digital health data with the LGG registry?  What assistance or support might be needed to contribute data?  Who would you expect to provide this support?  For IRB/researchers:  What rules or policies that exist to oversee this process?  Are there new rules or policies that would be needed?  T4: Protecting those who share digital health data: How might we need to protect privacy while making sure research participants are easily able to share their digital health data? What aspects of your data might you NOT want to share? Upon sharing data, what would be your expectation about who “owns” these data and what can be done with it?  To what extent would you expect security and privacy protections still apply after sharing through a third-party tool like Hugo Health? | *Date: 6/5/2022*  T1 // How has your experience been accessing digital health data to monitor you or your loved one’s health (eg. test results) – positive/neutral/negative?  Clinicians: What clinical health data do you share with patients? Do you provide them with specific guidance or tips? #BTSM  T2 // What kind of data do you wish you had access to? Is there any info you can't see or share w/ loved ones, other doctors, or research studies that would help in decision making/health monitoring?  Clinicians: Is there any health data you wish your patients had access to? #BTSM  T3A // Patients and care partners: Do you have any concerns with online health data? Is there anything that would make you feel more comfortable knowing your health info would be safe if you wanted to share it with a loved one, another doctor, or research study? #BTSM  T3B // Clinicians: Does the topic of health data privacy come up now that so many things are online? How do you talk to your patients and their care partners about it? #BTSM  T4 // If it was secure, would you be more likely to share health information with health partners, other doctors, or research studies? What kind of information or help would you need to share health data?  Clinicians: How might you help patients share their health data? #BTSM | *Dates: 9/8/2022 – 9/11/2022. Note: During this time frame, posts included both the data collection and return-of-results theme. For posts formatted as polls, both poll responses and additional comments were included in the analysis.*  Poll: How do you feel about sharing your medical records/personal health information for research purposes? Check out this link for more on this subject: https://www.youtube.com/watch?v=kt9u5omGwsY  a) I must be guaranteed full anonymity. None of my information is identifiable.  b) This is not a topic I have given a lot of thought to.  c) I trust the safeguards that are in place that protect my privacy.  d) The research should be relevant to a condition I have.  Post: How do you feel about using health apps that make it easy to connect your personal health information to research/studies? Is it for the good, or is it creepy? [Image: apps used to access or collect personal health information]  Poll: After surgery did you get a pathology report with your tumor’s IDH mutations and co-deletion status?”  a) Yes, but I’m not sure what it means  b) Yes, and my doctor used it to plan my treatment  c) Not that I know of, but I wish I did  d) I’m not sure.  Poll: It can take a long time for research to have findings to share! But when we do, how would you like learn about the results?  a) YouTube video,  b) a live “town hall” event from the research team  c) a website with plain language summaries of the findings  d) links to open access (free) scientific papers in journals |
| Return of results messages, materials, and protocols | *Dates: 6/29/2022, 6/30/2022*  T1: Tumor Recurrence: Have you ever had a recurrence of your tumor? Or, as a care partner, friend, or clinician, known someone who has? What have you noticed is different between the first tumor and the recurrence (e.g., symptoms, treatment strategies)?  T2: b. Glioma Tumor Reports:  Patients/CPs: Have you ever received a biomarker or genetics report on your tumor(s)? What types of information do these reports typically include, in your experience? What was your experience trying to understand what it meant for you or how your tumor might react to treatment?  Clinician or researcher, do you provide these reports to patients? How do you help patients understand what they mean?  T3: Returning Individual Research Results: The LGG Registry will return individual genetic testing results (research only; not certified as clinical findings) to people with recurrent tumors who participate in the OPTIMUM study. This will include both a tumor report and a report on other types of genetic risk. What kind of support or education might help research participants make sense of the results, or if they even want the results?  From a regulatory perspective, what policies or rules are in place to ensure research participants understand what individual research results mean for them?  T4: Returning Overall Research Results: At the end of the OPTIMUM study, we hope to have learned more about LGG tumors and why they return. What’s the best way to share the overall research findings with participants? With others like you? | *Date: 8/7/2022*  T1: Most brain tumors are at risk of recurrence.  Have you considered what steps you might take should you/your loved one’s tumor come back? If you’ve had a recurrence, what was different between the 1st tumor & the recurrence (e.g., symptoms, treatment strategies)? #BTSM  T2: Patients & Care Partners:  Pathology/biomarker reports are written for healthcare professionals.  What information have you seen in your tumor reports?  What approach might you take to better understand these reports and what they mean about a potential recurrence?  #BTSM  Clinicians & Researchers:  What experience do you have with sharing pathology/biomarker reports with patients?  How do you help patients w/recurrent tumors better understand what their reports mean? #BTSM  T3: Patients, Care Partners & Clinicians:  What kind of support/education might help you make better sense of your results or why your brain tumor came back?  What types of information about recurrence may feel overwhelming or challenging to understand?  #BTSM  T3: Researchers  Patients often get tumor results from doctors, but increasingly may also receive results directly from researchers.  What experiences do you have with returning results to participants? What rules are you required to follow? #BTSM  T4: What do YOU think is the best way for researchers to share overall/aggregate findings w/participants & neuro-oncology clinicians working to understand a recurrence? #BTSM  - Study website  - Email list  - YouTube channel  - Something else (comment below) |  |
