# Supplementary material for: Engagement Methods in Brain Tumor Genomic Research: Multimethod Comparative Study
Source: J Particip Med. 2025 Aug 21;17:e68852. doi: 10.2196/68852 (PMC12411796; doi:10.2196/68852)
Supplement: Multimedia Appendix 3 [file jopm_v17i1e68852_app3.docx]

**A Multi-Method Comparative Study of Engagement Methods in Brain Tumor Genomic Research**

Matthew DeCamp, MD, PhD;^1,2^ Juliana G. Barnard, MA;^3,4^ Carly Ritger, MS;^3^ Laura J. Helmkamp, MS;^3^ Anowara Begum, MPH;^3^ Sandra Garcia-Hernandez, MPH;^3^ Rudy Fischmann;^5^ Nestelynn Gay, MS;^5^ Ricardo Gonzalez-Fisher, MD, MPH;^5^ Kevin Johnson, PhD;^5,6^ Lindsay A. Lennox, BA;^7^ Guy R. Lipof;^5^ Jasmyn Ostmeyer, M.Ed;^5^ Ifeoma Perkins, MD,^2,8^ Laura Pyle, PhD;^4^ Liz Salmi, AS;^9^ Talia Thompson, PhD;^3,4^ Elizabeth B. Claus, MD, PhD;^10,11^ Roel Verhaak, PhD;^6,12^ Bethany M. Kwan, PhD^3,7^

**Affiliations**

^1^ Division of General Internal Medicine, University of Colorado School of Medicine, Aurora, CO

^2^ Center for Bioethics & Humanities, University of Colorado School of Medicine, Aurora, CO

^3^ Adult & Child Center for Outcomes Research & Delivery Science, University of Colorado Anschutz Medical Campus, Aurora, CO

^4^ Department of Pediatrics, University of Colorado School of Medicine, Aurora, CO

^5^ Low Grade Glioma Registry Research Advisory Council

^6^ Department of Neurosurgery, Yale School of Medicine, Yale University, New Haven, CT, USA

^7^ Department of Emergency Medicine, University of Colorado School of Medicine, Aurora, CO

^8^ Department of Pathology, University of Colorado School of Medicine, Aurora, CO

^9^ Department of Medicine, Beth Israel Deaconess Medical Center, Boston, MA

^9^ Children’s Hospital Colorado

^10^ Yale School of Public Health, New Haven, CT

^11^ Department of Neurosurgery, Brigham and Women’s Hospital, Boston, MA

^12^ Department of Neurosurgery, Amsterdam University Medical Center, Amsterdam, The Netherlands

**Corresponding author**: Bethany M. Kwan, PhD, MSPH; 12631 E 17^th^ Ave, Mail Stop C326, Aurora, CO 80045; [bethany.kwan@cuanschutz.edu](mailto:bethany.kwan@cuanschutz.edu)

Post-Engagement Survey 1

Low Grade Glioma Research Engagement Survey

Start of Block: Default Question Block

A Thank you for your interest in our survey. We are asking you to be in this research study because you recently discussed brain tumor genomic research topics (either online, or in person) with researchers from the Low Grade Glioma Registry. You might have participated in a "tweet chat" on Twitter with the brain tumor community, a Facebook discussion, or a live discussion over Zoom.

The purpose of this study is to understand what people like you think about engaging with genomic research. You can choose if you want to take the survey or not. The survey should take 10 minutes to complete. You will receive a $20 e-gift card for your time, within 10 days of completion. There is no direct benefit to you for participating. Your participation will not affect your medical care in any way. We will keep your information secure and confidential. If you provide us with your name, email address, or phone number there is a risk of loss of confidentiality. We will not share your answers with other researchers or your doctors in a way that they will know it’s you. 


We will be collecting your name, email, and/or phone number to verify survey responses, to provide compensation, and to invite you to future research opportunities. The data we collect will be used for this study but may also be important for future research. Your data may be used for future research or distributed to other researchers for future studies without additional consent, if information that identifies you is removed from the data. 


By completing this survey, you are agreeing to participate. 


 This research (Protocol 20-1001) was reviewed and declared Exempt by the Colorado Multiple Institutional Review Board (comirb@ucdenver.edu). It is being funded by the NIH/National Cancer Institute. If you have any questions, please contact Bethany Kwan at bethany.kwan@cuanschutz.edu.

B I am an adult between 18 and 89 years old who recently participated in at least one engagement activity with the Low Grade Glioma Registry, and I would like to complete this survey.

- Yes (1)
- No (2)

C Which of the following Low Grade Glioma Registry engagement activities did you participate in **most recently**? Please pick just the most recent. Please think about this engagement activity when you answer the following questions.

- May 2022 "Topic of the day" posts on the Oligodendroglioma/LGG Warriors Facebook group (6)
- Low Grade Glioma Research Advisory Council Meeting on 3/28/22 or 3/29/22 (5)
- #BTSM Tweet Chat on 3/6/2022 on "Understanding brain tumor genes" (2)
- I did not participate in any of these Low Grade Glioma Registry engagement activities recently (4)

Display This Question:

If I am an adult between 18 and 89 years old who recently participated in at least one engagement ac... = No

Or Which of the following Low Grade Glioma Registry engagement activities did you participate in mos... = I did not participate in any of these Low Grade Glioma Registry engagement activities recently

Q1 Thank you for your interest! You are not eligible to participate in this survey at this time.

Skip To: End of Survey If Thank you for your interest! You are not eligible to participate in this survey at this time. Displayed

| Page Break |  |
| --- | --- |

Q2 YOUR VIEWS OF MEDICAL RESEARCH 
Thank you for agreeing to participate in our survey! Your opinions matter. Let’s get started.

Q3 Before we ask you more about your most recent participation in an engagement activity with researchers from the Low Grade Glioma Registry, we want to learn what you think about medical research in general. Please rate your agreement with the following 4 statements:

|  | Strongly disagree (1) | Disagree (2) | Neutral (3) | Agree (4) | Strongly agree (5) |
| --- | --- | --- | --- | --- | --- |
| Doctors who do medical research care only about what is best for each patient. (1) |  |  |  |  |  |
| Doctors tell their patients everything they need to know about being in a research study. (2) |  |  |  |  |  |
| Medical researchers treat people like “guinea pigs.” (3) |  |  |  |  |  |
| I completely trust doctors who do medical research. (4) |  |  |  |  |  |

| Page Break |  |
| --- | --- |

Q4 YOUR IMPRESSIONS OF YOUR RECENT ENGAGEMENT ACTIVITY 
Now we want to learn more about the most recent engagement activity you had with researchers from the Low Grade Glioma Registry.

Q5 Please think about your most recent Low Grade Glioma Registry engagement activity as indicated above. We would like to learn about your preparation for this engagement activity. Please rate your agreement with the following 3 statements.

|  | Strongly disagree (1) | Disagree (2) | Neutral (3) | Agree (4) | Strongly agree (5) |
| --- | --- | --- | --- | --- | --- |
| The purpose of the activity was clearly explained. (1) |  |  |  |  |  |
| The supports I needed to participate were available (e.g., travel, child care, technology). (2) |  |  |  |  |  |
| I had enough information to contribute to the topic being discussed. (3) |  |  |  |  |  |

| Page Break |  |
| --- | --- |

Q6 Now we want to learn what you thought about the input you, and others, provided. Please rate your agreement with the following statements.

|  | Strongly disagree (1) | Disagree (2) | Neutral (3) | Agree (4) | Strongly agree (5) |
| --- | --- | --- | --- | --- | --- |
| I was able to express my views freely. (1) |  |  |  |  |  |
| I feel that my views were heard. (2) |  |  |  |  |  |
| A wide range of views on the topic were expressed. (3) |  |  |  |  |  |
| This activity included diverse participants from different backgrounds and walks of life. (4) |  |  |  |  |  |

| Page Break |  |
| --- | --- |

Q7 The next few statements assess what you think will happen as a result of the recent engagement activity. Please rate your agreement with the following statements.

|  | Strongly disagree (1) | Disagree (2) | Neutral (3) | Agree (4) | Strongly agree (5) |
| --- | --- | --- | --- | --- | --- |
| I feel that the input provided through this activity will be considered by the organizers. (1) |  |  |  |  |  |
| The activity achieved its stated objectives. (2) |  |  |  |  |  |
| I understand how the input from this activity will be used. (3) |  |  |  |  |  |
| I think this activity will make a difference. (4) |  |  |  |  |  |
| As a result of my participation in this activity, I am better informed about the Low Grade Glioma Registry. (5) |  |  |  |  |  |
| As a result of my participation in this activity, I have greater trust in the researchers who are leading the Low Grade Glioma Registry. (6) |  |  |  |  |  |
| As a result of my participation, I think others will be more likely to participate in the Low Grade Glioma Registry. (7) |  |  |  |  |  |

| Page Break |  |
| --- | --- |

Q8 Next, we'd like to learn what you thought about this engagement activity, overall. Please rate your agreement with the following statements.

|  | Strongly disagree (1) | Disagree (2) | Neutral (3) | Agree (4) | Strongly agree (5) |
| --- | --- | --- | --- | --- | --- |
| Overall, I was satisfied with this activity. (1) |  |  |  |  |  |
| This activity was a good use of my time. (2) |  |  |  |  |  |

Q9 How much time did you spend preparing for and participating in this engagement activity?

- Less than one hour (1)
- 1 to 2 hours (2)
- 3 to 4 hours (3)
- More than 4 hours (4)

Q10 Did you have any direct personal expenses related to preparing for or participating in this engagement activity? For example, this might include child or other dependent care, special internet access, transportation or other travel expenses, or new technology devices needed for this activity.

- Yes (1)
- No (2)
- I'm not sure (3)

Display This Question:

If Did you have any direct personal expenses related to preparing for or participating in this engag... = Yes

Q11 If yes, please tell us about these expenses.

________________________________________________________________

________________________________________________________________

________________________________________________________________

________________________________________________________________

________________________________________________________________

Display This Question:

If Did you have any direct personal expenses related to preparing for or participating in this engag... = Yes

| 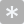 |
| --- |

Q12 If you had to put a dollar amount on these expenses, how much would you say you spent to participate in this engagement activity?

________________________________________________________________

| Page Break |  |
| --- | --- |

Q13 YOUR IMPRESSIONS OF THE RESEARCHERS LEADING THE ENGAGEMENT:

| 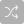 |
| --- |

Q14 Which of the following Low Grade Glioma Registry researchers do you remember being part of this engagement activity, if any? Select all that apply.

- Bethany Kwan, from the University of Colorado (1)
- Elizabeth Claus, from Yale University and the Brigham & Women's Hospital (2)
- Roel Verhaak, from the Jackson Laboratory (3)
- Liz Salmi, from Beth Israel Deaconess Medical Center (6)
- Kevin Johnson, from the Jackson Laboratory (4)
- Juli Barnard, from the University of Colorado (7)
- Jenna Reno, from the University of Colorado (8)
- Carly Ritger, from the University of Colorado (9)
- I don't recall any Low Grade Glioma Registry researchers being part of this activity (5)

| Page Break |  |
| --- | --- |

Q15 Now please think about your experience with the people who led the engagement activity. For these next few items, please rate how well you think the people who led the engagement did:

|  | N/A Don't Know (1) | Poor (2) | Fair (3) | Good (4) | Very Good (5) | Excellent (7) |
| --- | --- | --- | --- | --- | --- | --- |
| The focus is on problems important to the community. (1) |  |  |  |  |  |  |
| All partners assist in establishing roles and related responsibilities for the partnership. (2) |  |  |  |  |  |  |
| Community-engaged activities are continued until the goals (as agreed upon by all partners) are achieved. (3) |  |  |  |  |  |  |
| The partnership adds value to the work of all partners. (4) |  |  |  |  |  |  |
| The team builds on strengths and resources within the community or patient population. (5) |  |  |  |  |  |  |
| All partners’ ideas are treated with openness and respect. (6) |  |  |  |  |  |  |
| The partnership’s processes support trust among all partners. (7) |  |  |  |  |  |  |
| Mutual respect exists among all partners. (8) |  |  |  |  |  |  |

| Page Break |  |
| --- | --- |

| Page Break |  |
| --- | --- |

Q16 ABOUT YOU 
These last few questions are about you. We want to learn more about the people who are engaging with researchers on this project.

Q17 How old are you?

- Under 18 years old (1)
- 18-24 years old (2)
- 25-29 years old (3)
- 30-39 years old (4)
- 40-49 years old (5)
- 50-59 years old (6)
- 60-69 years old (7)
- 70-79 years old (8)
- 80-89 years old (9)
- Over 90 years old (10)

Q18 How do you describe yourself?

- Man (1)
- Woman (2)
- Non-binary / third gender (3)
- Prefer to self-describe (4) __________________________________________________
- Prefer not to say (5)

Q19 Are you Spanish, Hispanic, or Latino or none of these?

- Yes (1)
- None of these (2)

Q20 Choose one or more races that you consider yourself to be:

- White (1)
- Black or African American (2)
- American Indian or Alaska Native (3)
- Asian (4)
- Native Hawaiian or Pacific Islander (5)
- Other (6) __________________________________________________

Q21 What is the highest level of school you have completed or the highest degree you have received?

- Less than high school degree (1)
- High school graduate (high school diploma or equivalent including GED) (2)
- Some college but no degree (3)
- Associate degree in college (2-year) (4)
- Bachelor's degree in college (4-year) (5)
- Master's degree (6)
- Doctoral or professional degree (PhD, JD, MD, others) (7)

Q22 Information about income is very important to understand.  Would you please give your best guess? Please indicate the answer that includes your entire household income in (previous year) before taxes.

- Less than $50,000 (1)
- $50,000 to $99,999 (2)
- $100,000 or more (3)
- I prefer not to say (4)

Q23 What type of health insurance coverage do you currently have, if any? Select all that apply.

- Private insurance provided by my or a partner or parent's employer (1)
- Private insurance purchased on my own (e.g., through HealthCare.gov or a state insurance marketplace) (2)
- Medicare (insurance through the US federal government for people over age 65 or with disabilities) (3)
- Medicaid (insurance through state government for people who meet income qualifications) (4)
- TriCare or other insurance provided to US veterans (5)
- Universal healthcare (10)
- Not sure (6)
- Other type of insurance, please specify (7) __________________________________________________
- Prefer not to say (8)

| 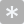 |
| --- |

Q24 What is your ZIP code?

________________________________________________________________

| 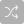 |
| --- |

Q25 What type(s) of stakeholder perspective do you bring to brain tumor genomic research? Select all that apply.

- I have personally been diagnosed with a brain tumor. (1)
- I am a care partner (such as a family member or friend) for someone who has been diagnosed with a brain tumor. (2)
- I am a researcher who studies brain tumors or topics related to brain tumors. (3)
- I am a health care provider who cares for people with brain tumors. (4)
- I am a representative of an advocacy organization or other service organization that addresses issues important to people with brain tumors. (5)
- I represent an agency or organization that provides funding for brain tumor research. (6)
- I am a community member with a general interest in brain tumors and/or genomic research. (7)

Display This Question:

If What type(s) of stakeholder perspective do you bring to brain tumor genomic research? Select all... = I have personally been diagnosed with a brain tumor.

Q26 Have you personally ever been diagnosed with a brain tumor? If so, what was the most recent brain tumor diagnosis you have received?

- Astrocytoma (1)
- Oligodendroglioma (2)
- Other low grade glioma (3)
- Glioblastoma (4)
- Other high grade glioma (5)
- Other glioma (6)
- Other brain tumor (7)
- No, I have not personally been diagnosed with a brain tumor (8)

| Page Break |  |
| --- | --- |

Q27 Verification and Gift Cards

Display This Question:

If RecipientEmail Is Empty

Q28 So that we can verify that you are someone who participated in an engagement activity, please tell us your name. (First and Last)

________________________________________________________________

Q29 Would you be willing to be contacted for additional Low Grade Glioma Registry research opportunities? For instance, future research opportunities may include additional surveys or focus groups. There would be additional compensation for other studies. If you are willing to be contacted, please indicate that here:

- Yes (3)
- No (4)

Skip To: Q33 If Would you be willing to be contacted for additional Low Grade Glioma Registry research opportunit... = No

| Page Break |  |
| --- | --- |

Q30 Please indicate the best way to reach you:

- Phone - call (1)
- Phone - text (2)
- Email (3)

| Page Break |  |
| --- | --- |

Display This Question:

If Please indicate the best way to reach you: = Phone - call

Or Please indicate the best way to reach you: = Phone - text

| 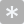 |
| --- |

Q31 What is your preferred phone number?

________________________________________________________________

Display This Question:

If Please indicate the best way to reach you: = Email

| 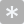 |
| --- |

Q32 What is your preferred email address for future research opportunities?

________________________________________________________________

| Page Break |  |
| --- | --- |

Q33 Thank you for completing our survey about engagement in brain tumor genomic research. Once we have verified responses, we will use the information below to send you a link to select and redeem your $20 e-gift card on a website called Tango Card. An e-gift card is electronic and will be delivered in an email; it can be printed and used in store or used as a code that can be used online or shown in store. Remember to check your junk or spam folder in your email if you are having trouble finding the information on how to redeem your e-gift card. We will send out the gift card within 10 days. If you have not received your gift card by then, please email us at OPTIMUM.EOU@cuanschutz.edu. 


Note, you may only complete the survey once. If responses appear to be duplicates or were not answered seriously, we may not send you a gift card. Skip this question if you do not wish to receive a gift card.

Q40 What email address should the e-gift card be sent to?

________________________________________________________________

End of Block: Default Question Block
