# Supplementary material for: Engagement Methods in Brain Tumor Genomic Research: Multimethod Comparative Study
Source: J Particip Med. 2025 Aug 21;17:e68852. doi: 10.2196/68852 (PMC12411796; doi:10.2196/68852)
Supplement: Multimedia Appendix 5 [file jopm_v17i1e68852_app5.pdf]

Online Resource 5. CHERRIES Checklist

Category	*Checklist Item*	*Explanation*
**Design**		
	Describe survey design	Convenience sample of all included engagement participants across three methods of engagement: advisory panel, Facebook, and Twitter (now X)
**IRB (Institutional Review Board) approval and informed consent process**		
	IRB approval	This study was approved by the Colorado Multiple Institutional Review Board (COMIRB protocol #20-1001) as exempt human subjects research.
	Informed consent	Standard survey consent. During recruitment and at the time of survey, participants were told about rationale for the study (i.e., to understand and improve how engagement gets done), the time requirement (10 minutes), compensation, and the potential for data to be deidentified for future research. Participants were given the option to fill out the survey anonymously.
	Data protection	Data were stored and accessed only from secure, password protected network drives at the University of Colorado.
**Development and pre-testing**		
	Development and testing	The survey was developed and pre-tested by 5 people (one community, 4 academic) before launch in the Qualtrics platform.

Category	*Checklist Item*	*Explanation*
**Recruitment process and description of the sample having access to the questionnaire**		
	Open survey versus closed survey	Closed – only engagement participants were eligible.
	Contact mode	RAC members received an email invitation via Qualtrics. For Facebook and Twitter, the group administrators posted an announcement about the opportunity to participate in a survey, indicating interested participants should direct message the study lead to receive a link to the survey. Engagement participants also received direct messages from the study lead or group administrator inviting them to complete the survey
	Advertising the survey	This was targeted survey, so open advertising was not used.
**Survey administration**		
	Web/E-mail	Survey was sent via email, and responses were automatically collected via Qualtrics.
	Context	N/A – individuals were directed by invitation to the survey website.
	Mandatory/voluntary	Voluntary
	Incentives	$20 reimbursement
	Time/Date	March – September 2022
	Randomization of items or questionnaires	N/A – randomization was not done.

Category	*Checklist Item*	*Explanation*
	Adaptive questioning	N/A – no adaptive questioning was needed, given the brief time requirements of the instrument.
	Number of Items	Varied. The number per page was optimized to avoid scrolling and for mobile device access.
	Number of screens (pages)	8
	Completeness check	Items were
	Review step	No summary or individual responses was given, but participants were allowed to navigate to change answers.
**Response rates**		
	Unique site visitor	N/A – we did not need to track this given the nature of our targeted recruitment.
	View rate (Ratio of unique survey visitors/unique site visitors)	N/A – we did not need to track this given the nature of our targeted recruitment.
	Participation rate (Ratio of unique visitors who agreed to participate/unique first survey page visitors)	See description in text. As a pilot exploratory survey, we did not calculate a participation or response rate.
	Completion rate (Ratio of users who finished the survey/users who agreed to participate)	We estimate a participation rates as 52/154 engagement 1 (33.8%) and 40/187 (21.3%) post engagement 4.

Category	*Checklist Item*	*Explanation*
**Preventing multiple entries from the same individual**		
	Cookies used	Each individual received a unique link
	IP check	IP check was not used for our study.
	Log file analysis	N/A
	Registration	Because we offered participants anonymity we did not require a login.
**Analysis**		
	Handling of incomplete questionnaires	Were only completed questionnaires analyzed? Were questionnaires which terminated early (where, for example, users did not go through all questionnaire pages) also analyzed?
	Questionnaires submitted with an atypical timestamp	Some investigators may measure the time people needed to fill in a questionnaire and exclude questionnaires that were submitted too soon. Specify the timeframe that was used as a cut-off point, and describe how this point was determined.
	Statistical correction	N/A for this exploratory study.
